# Post-Translational Modifications to Cysteine Residues in Plant Proteins and Their Impact on the Regulation of Metabolism and Signal Transduction

**DOI:** 10.3390/ijms25189845

**Published:** 2024-09-12

**Authors:** Charlie Boutin, Camille Clément, Jean Rivoal

**Affiliations:** Institut de Recherche en Biologie Végétale, Université de Montréal, 4101 Rue Sherbrooke est, Montréal, QC H1X 2B2, Canada; charlie.boutin@umontreal.ca (C.B.); camille.clement@umontreal.ca (C.C.)

**Keywords:** post-translational modification, cysteine, regulation, signal transduction, metabolism, thiol, redox modification

## Abstract

Cys is one of the least abundant amino acids in proteins. However, it is often highly conserved and is usually found in important structural and functional regions of proteins. Its unique chemical properties allow it to undergo several post-translational modifications, many of which are mediated by reactive oxygen, nitrogen, sulfur, or carbonyl species. Thus, in addition to their role in catalysis, protein stability, and metal binding, Cys residues are crucial for the redox regulation of metabolism and signal transduction. In this review, we discuss Cys post-translational modifications (PTMs) and their role in plant metabolism and signal transduction. These modifications include the oxidation of the thiol group (*S*-sulfenylation, *S*-sulfinylation and *S*-sulfonylation), the formation of disulfide bridges, *S*-glutathionylation, persulfidation, *S*-cyanylation *S*-nitrosation, *S*-carbonylation, *S*-acylation, prenylation, CoAlation, and the formation of thiohemiacetal. For each of these PTMs, we discuss the origin of the modifier, the mechanisms involved in PTM, and their reversibility. Examples of the involvement of Cys PTMs in the modulation of protein structure, function, stability, and localization are presented to highlight their importance in the regulation of plant metabolic and signaling pathways.

## 1. Introduction

Protein post-translational modifications (PTMs) are chemical or enzymatic modifications of proteins that can affect various aspects of protein activity through changes in structure, function, regulation, localization, interactions, or stability, to name a few examples [1]. A large number of PTMs have been described in plants, which is similar to the situation for other living organisms [1,2,3,4,5]. Due to their sessile lifestyle, PTMs are especially important for plants in which they serve as efficient regulatory mechanisms, allowing rapid and often reversible cellular responses to changes in homeostasis, as well as adjustments to alterations in metabolism, physiology, or external stimuli [2,5,6]. In plants, one of the most widespread consequences resulting from modifications in their biotic or abiotic environment is an alteration in the cell redox status [7,8,9,10]. Such changes govern a variety of redox signaling events that are involved in adjusting plant metabolism or signaling pathways [7,9,11,12]. Redox signaling notably occurs via the oxidative modification of Cys residues in proteins [13].

Cys is thought to be a comparatively recent addition to the genetic code [14] and is involved in important structural and functional regions of proteins [15,16]. Since two codons translate to Cys, the latter should theoretically represent 3.3% of the amino acids in proteins [17]. However, it is underrepresented in organisms from all kingdoms of life [17]. Interestingly, the Cys content in proteins increases with the complexity of the organisms, ranging from 0.4 to 0.5% in Archae to around 2.3% in mammals [17,18]. When it comes to photosynthetic organisms, analyses show a similar trend, with a Cys content ranging from ~1.1% in cyanobacteria to ~1.5% in green algae and ~1.9% in land plants [17,18,19]. The vast majority of proteins contain Cys. For instance, more than 92% of total plant protein sequences contain at least one Cys, with a median of six residues/protein [18]. In most organisms, the Cys distribution in protein sequences is also peculiar, with a preference for the CXXC sequence pattern [17]. The CXXC motif is usually found in oxidoreductase and metal-binding domains [17]. However, in contrast to other organisms, plant proteins do not exhibit a high level of representation of this specific motif [17]. Cys also displays a distinctive conservation pattern. In most proteins, Cys is either more than 90% conserved, or less than 10% conserved, indicating a strong selective pressure to both maintain important functional Cys and remove other ones [20]. The negative selection of Cys residues appears to be stronger for isolated Cys present on protein surfaces [20].

Cys has physico-chemical properties that are unique among all protein amino acids. Its side chain carries a thiol group (-SH), which can deprotonate as a consequence of various interactions with its environment. This loss of a proton generates a thiolate (-S^−^), increasing the nucleophilicity of the side chain [16]. This is a key determinant in Cys reactivity. The average p*K*_a_ of protein Cys residues exposed to the solvent is around 7.5, a much lower value than that of buried Cys (around 9.5). This low p*K*_a_ contributes to the fact that surface Cys are much more reactive [20]. Indeed, all protein Cys residues are not equally reactive. With a p*K*_a_ value of 8.45 found for free Cys thiols, most iterations of Cys would be expected to exist in their protonated form [21]. However, the protein microenvironment can significantly affect Cys thiols’ p*K*_a_. For instance, basic amino acids, such as His, Arg or Lys, and as metal ions adjacent to Cys residues tend to lower the p*K*_a_ of Cys thiols, stabilizing its thiolate form and promoting its reactivity [22,23]. The end-positioning of Cys residues on α-helices is another factor that affects their p*K*_a_. It has long been known that α-helices behave as dipoles [24]. Studies of the thioredoxin superfamily have shown that the dipole properties of an α-helix, together with the localization of a Cys at the N-terminus of the helix, contribute to lowering the thiol p*K*_a_, thereby enhancing its reactivity [25,26]. Another way by which the protein microenvironment might increase the reactivity of a thiol group is by decreasing the activation energy during the transition state of the reaction involving the thiol. This has been demonstrated in the case of mechanistic studies of thiol/disulfide exchange [27,28]. In enzyme-catalyzed reactions, contrary to a polar solvent, the hydrophobic environment provided by the protein does not stabilize the reactants in relation to the reaction transition state. This process reduces the activation energy required for the reaction to proceed, allowing a faster reaction rate [28].

The reactivity of thiols is a crucial factor responsible for the involvement of Cys residues in multiple facets of protein function, including folding, catalysis, proteostasis, and signal transduction [29]. The Cys sulfur atoms possess oxidation states ranging from −2 to + 4 [30,31], allowing a variety of redox post-translational modifications (PTMs). Disulfide type reversible modifications of Cys residues, such as disulfide bridge or mixed disulfide with low-molecular-weight thiols, are often referred to as redox or thiol switches [29,32]. These play an important role in the modulation of protein activity, function, and localization in response to external stimuli. Redox signaling can involve reactive oxygen species (ROS), reactive nitrogen species (RNS), reactive carbonyl species (RCS), and reactive sulfur species (RSS) [33]. The PTMs of Cys residues include *S*-sulfenylation, *S*-sulfinylation and *S*-sulfonylation, disulfide bridge (S-S) formation, *S*-glutathionylation, persulfidation, *S*-cyanylation, *S*-nitrosation, *S*-carbonylation, *S*-acylation, prenylation, CoAlation, and thiohemiacetal formation. An analysis of the literature shows the increasing complexity of Cys modifications in plants and reveals evidence of a growing recognition of the importance of these PTMs in the regulation of metabolism and signal transduction. For reference, a searchable database catalogs modified proteins and PTM sites in several model plants (Plant PTM Viewer 2.0, https://www.psb.ugent.be/webtools/ptm-viewer/, (accessed on 30 July 2024)) [4]. Another database, based on a deep learning framework, was recently established to facilitate protein Cys modifications in eukaryotes (pCysMod, http://pcysmod.omicsbio.info/, (accessed on 30 July 2024)) [34]. Unsurprisingly, many plant proteins are identified as targets for multiple PTMs. Indeed, since multiple molecules present in the same cell compartment at the same time are able to interact with reactive thiols, multiple Cys modifications can compete with each other in vivo, adding a layer of complexity in the understanding of Cys-mediated signaling in plant cells [33,35,36,37].

The aim of this review is to provide a comprehensive survey of the various PTMs of Cys residues identified in plants. For each modification, we evaluate the current state of knowledge regarding mechanisms facilitating PTM and its reversion, whether spontaneous or enzymatic. For each PTM, examples of targets involved in metabolism and/or signal transduction are identified and the implications of the modification in the regulation of these targets are critically discussed in relation to their function. In some cases, gaps in knowledge and possible future avenues of research on Cys PTMs in plants are also identified.

## 2. *S*-Sulfenylation

Under normal conditions, aerobic metabolic processes, such as photosynthesis and respiration, constantly produce low levels of ROS as by-products. ROS are present in the cell in different forms, such as O_2_^●−^ (superoxide radical), H_2_O_2_ (hydrogen peroxide), and ^●^OH (hydroxyl radical) [38]. Various biotic and abiotic stresses can disrupt redox homeostasis by promoting significant increases in ROS, leading to oxidative stress. Basal and stress-induced ROS production and detoxification in plants have been extensively reviewed and the reader is therefore invited to consult the relevant publications and references within [39,40,41,42,43,44,45,46,47,48]. ROS greatly differ in their reactivity, diffusion rate, and concentration in cells [38]. They can also cause a range of reversible and irreversible damage to lipids, DNA, and proteins, altering their function in cells [41,42]. Although they were initially thought to be exclusively toxic molecules that lead to oxidative distress, it is now widely recognized that ROS can also generate oxidative eustress, or ‘good stress’ [49], via essential signaling functions [33,37,38,50].

### 2.1. S-Sulfenylation Is Promoted by Oxidative Conditions and Is a Stepping-Stone towards Other Cys Redox PTMs

**Figure 1:**
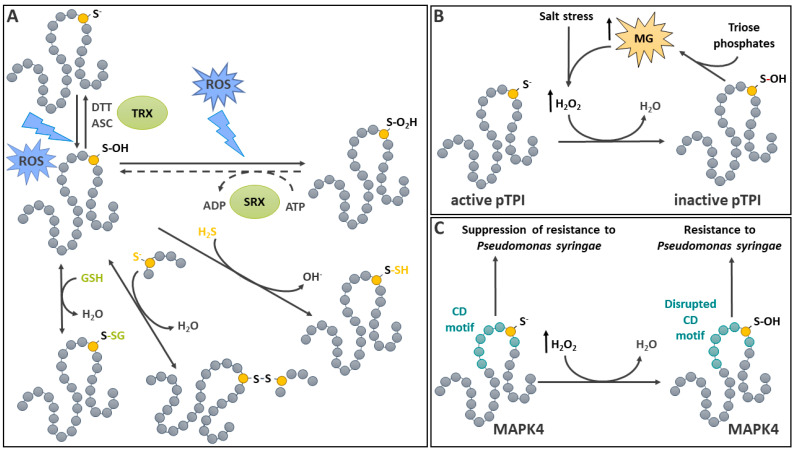
Protein *S*-sulfenylation in plants. (**A**): The transition of Cys residues from a thiolate state to an *S*-sulfenylated state. A Cys residue (yellow dot) in a deprotonated state (thiolate, S^−^) (top left corner) undergoes oxidation to its sulfenic acid form (S-OH) upon exposure to oxidative conditions caused by reactive oxygen species (ROS). Reductants such as dithiothreitol (DTT), ascorbate (ASC), and thioredoxin (TRX) have been shown to reverse this reaction. The *S*-sulfenylated form allows further Cys modifications, such as the generation of a reversible mixed disulfide using glutathione (GSH) to yield *S*-glutathionylation (S-SG); a reaction with a protein Cys in thiolate form to generate a reversible disulfide bridge (S-S); a reaction with H_2_S to generate a persulfidation (S-SH); and a higher degree of oxidation towards the sulfinic acid form (SO_2_H). The latter reaction can be reversed with an ATP-dependent sulfiredoxin (SRX). (**B**): The inhibition of plastidial triose phosphate isomerase (pTPI) by *S*-sulfenylation. Under high-H_2_O_2_ conditions created by salt stress, pTPI becomes inactive due to *S*-sulfenylation. The inability of pTPI to process triose phosphates leads to the formation of methylglyoxal, which acts as a promoter of H_2_O_2_ formation. (**C**): The regulation of MAPK4 by *S*-sulfenylation. MAPK4 is a negative regulator of resistance to *Pseudomonas syringae*. Under normal conditions, the protein is active and suppresses resistance to the pathogen. Under oxidative stress conditions, a Cys residue of the protein’s Common Docking (CD) motif becomes *S*-sulfenylated, thereby disrupting MAPK4 function and enabling resistance to the pathogen. See the text for additional details.

Among ROS, H_2_O_2_ has the longest half-life and the highest capacity for diffusion [41], which makes it highly suitable for redox signaling. Indeed, H_2_O_2_ acts as a second messenger in plants by diffusing in cells and across membranes via aquaporins, thereby allowing both autocrine and paracrine signaling [45]. H_2_O_2_ is relatively stable and its reaction with reduced free Cys or glutathione (GSH) is slow compared to some other ROS and RCS [49]. H_2_O_2_ generally reacts more easily with protein Cys, but its reactivity for thiol oxidation is highly dependent on a favorable protein microenvironment reducing the activation energy [49]. This dependency on protein structure for reactivity determines the specificity of H_2_O_2_-mediated redox signals [49]. Thiolates, which are more reactive than thiols towards H_2_O_2_, can perform nucleophilic attacks on H_2_O_2_, causing reversible two-electron oxidation to sulfenic acid (-SOH) [51], potentially altering enzyme function and activity (Figure 1) [42]. It is noteworthy that, in addition to H_2_O_2_, natural or artificial hydroperoxides and peroxynitrite can also cause thiol oxidation to sulfenic acid [31,52]. The latter is usually considered highly unstable and acts as an intermediate towards several Cys redox PTMs (Figure 1A), including, as discussed below, *S*-sulfinylation, *S*-sulfonylation, *S*-glutathionylation, S-S formation, or persulfidation [22]. As discussed in Section 3 below, there is also an enzymatic pathway responsible for the generation of *S*-sulfenylated Cys. This occurs in instances where *S*-sulfinylated Cys can be reduced using sulfiredoxin (SRX) [53]. The stability of the *S*-sulfenylated Cys is mainly determined by its molecular environment. The improvement of sulfenic acid’s stability is caused by decreased solvent accessibility, the absence of a proximal Cys that could induce the formation of a S-S, and the stabilization of the sulfenate using an H-bond network with adjacent amino acids [22]. In addition, sulfenic acid has unique reactivity since it can act both as a nucleophile and an electrophile [31]. For instance, the nucleophilic reactions of sulfenic acid include its overoxidation to sulfinic acid [31]. For this, sulfenic acid performs a nucleophilic attack on H_2_O_2_, leading to irreversible sulfinic and sulfonic forms of oxidation, as discussed below. Electrophilic sulfenic acid reactions lead, for example, to Cys persulfidation, which involves a reaction with H_2_S and cannot occur with a non-oxidized thiolate (see Section 6 below, [54]). Additionally, sulfenic acid can react with a thiol to create an intramolecular or intermolecular S-S or a mixed disulfide [31] (see the S-S and *S*-glutathionylation sections below). The ability of sulfenic acid to act as an electrophile has also been exploited by using its reactivity to 5,5-dimethyl-1,3-cyclohexanedione (dimedone). This highly selective reaction has been used to develop chemoselective dimedone-based probes, enabling the detection of sulfenylated proteins in cells [55]. More recently, a more reactive benzo[c][1,2]thiazine-based (BTD) probe [56] was used to identify the *Arabidopsis thaliana* (Arabidopsis) sulfenome [57]. Chemoselective methods for surveying and identifying the different levels of Cys thiol oxidation have been recently reviewed [58].

### 2.2. S-Sulfenylation Is a Reversible Primary Cys Oxidation

The reversion of Cys thiol oxidation from sulfenic acid back to the thiol form is possible (Figure 1). This can be achieved in vitro using a variety of reducing agents, such as dithiothreitol (DTT), arsenite [31], or ascorbate in the case of 1-Cys peroxiredoxins (PRXs) [59,60]. The pool of ascorbate is normally highly reduced in plants [61]. Thus, the in vitro activity of ascorbate in the reduction of Cys sulfenic acid may have some relevance in vivo, although this remains to be seen. Nevertheless, the role of ascorbate in this process has been known for some time in animals [62]. The reduction of sulfenic acid can also occur via other mechanisms [63]. Methionine sulfoxide reductase B1 (MSRB1) is a 1-Cys reductase involved in the reduction of Met sulfoxide back to Met, allowing proteins that carry oxidized Met to return to their basic state. In the process, the catalytic Cys of MSRB1 becomes oxidized to sulfenic acid. The reaction of the *S*-sulfenylated catalytic Cys with GSH leads to its *S*-glutathionylation (see Section 5 for mechanistic details). The *S*-glutathionylated Cys can then be reduced back to its thiol form using glutaredoxin (GRX) [63]. The study demonstrated that GRXC4 and GRXS12 can carry out the reaction in vitro. In addition to GSH, TRX can act as an electron donor for the reduction of *S*-sulfenylated Cys. While the latter work showed a lack of capacity for TRX*h*1 to reduce *S*-sulfenylated MSRB1 [63], MSRB2 could be used as a substrate. A subsequent study provided strong evidence that *S*-sulfenylated MSRB1 could be reduced using TRX CDSP32 under physiological conditions in a process that involved the formation of an S-S [64]. More recently, the enzymatic reduction of protein sulfenic acid has also been demonstrated in vitro for EF-Tu, a redox-sensitive chloroplastic translation elongation factor of Arabidopsis [65]. The study identified that TRX *f*1 mediated the reduction of *S*-sulfenylated Cys^149^. Thus, various pathways may contribute to the conversion of Cys sulfenic acid into its thiol form, and much remains to be investigated concerning the possible differences in the efficiency of the various actors involved in the process.

### 2.3. S-Sulfenylation as a Redox-Control Mechanism in Plant Primary Metabolism

ROS-mediated oxidation modulates the activity of many enzymes involved in metabolism and stress responses. Cellular oxidative conditions, as discussed above, are conducive to protein *S*-sulfenylation. A survey of the Arabidopsis cell culture, sulfenylome, revealed that more than 1000 proteins were *S*-sulfenylated in response to treatments with H_2_O_2_ (up to 400 µM), with an average of 1.5 modifications/protein [57]. The study noted a particularly significant enrichment in protein classes belonging to important metabolic pathways, while several *S*-sulfenylated sites were predicted or proved to have functional importance. Among these in vivo targets, cytosolic glyceraldehyde-3-phosphate dehydrogenase (GAPDH) had been previously shown to be strongly inhibited by H_2_O_2_ [66]. In the case of GAPDH, *S*-sulfenylation can also lead to inhibitory *S*-glutathionylation (see below). A decrease in cytosolic GAPDH activity, such as that documented in an *Arabidopsis knockout* line, leads to decreased ATP and tricarboxylic cycle intermediate pools [67]. This is consistent with the fact that oxidative stress generally inhibits several aspects of respiratory metabolism [6]. Moreover, in plants and other systems, the oxidative inhibition of triose phosphate metabolism enzymes in glycolysis has been linked to a redirection of C flux to the oxidative pentose phosphate pathway (OPPP) involved in NADPH generation. [6,68]. This can be used by enzymes in the redoxin family in order to restore redox homeostasis. The evolutionary conservation of this redox-controlled metabolic switch between glycolysis and the OPPP may indicate the strategy has an ancient origin.

Lately, there have been other examples illustrating the potential for Cys *S*-sulfenylation to act as a regulatory mechanism for metabolic enzymes in the chloroplast. An initial investigation showed that the plastidial triosephosphate isomerase in Arabidopsis is inhibited by Cys^74^ *S*-sulfenylation, resulting from H_2_O_2_ accumulation induced by salt stress (Figure 1B) [69]. A consequence of this inhibition was an accumulation of methylglyoxal [69], which is known to promote H_2_O_2_ formation [70], thus creating a feedback loop. More recently, the examination of the redox properties of plastidial NAD-dependent malate dehydrogenase revealed four redox-active Cys [71]. It was determined that, following in vitro oxidative treatment, a great proportion of Cys^129^ was *S*-sulfenylated. This residue was further found to be responsible for the reversible oxidative inhibition of the enzyme using a directed mutagenesis study. Interestingly, reduced or oxidized nicotinamide adenine dinucleotide cofactors offered a relatively high level of protection against plastidial NAD-dependent malate dehydrogenase inhibition [71]. This proposed *S*-sulfenylation regulatory mechanism could impact stromal compartment dicarboxylate metabolism in a redox-dependent manner. However, this remains to be further established using in vivo approaches.

### 2.4. Involvement of Protein S-Sulfenylation in Stress Signal Transduction

In the past years, several proteomic surveys have illustrated the importance of *S*-sulfenylation-dependent mechanisms in plant stress signaling [57,72,73,74]. Current approaches to study this topic take advantage of the above-mentioned dimedone-based sulfenate probes in vivo. A recent publication illustrates the role played by the Respiratory Burst Oxidase Homolog (RBOH) in protein *S*-sulfenylation during the response to pathogens [74]. The RBOH is a plasma membrane-localized NADPH oxidase that generates O_2_^●−^ in the apoplast [75]. H_2_O_2_ is then formed upon the detoxification of O_2_^●−^ by a superoxide dismutase present in the extracellular space [76]. RBOH is an important player involved in the recognition of Pathogen-Associated Molecular Patterns (PAMPs) [77]. Upon recognition by pathogen-derived signals, plasma membrane receptors trigger a phospho-relay signaling cascade that leads to the activating phosphorylation of RBOH by Ca^2+^-dependent protein kinases and Botrytis-induced kinase 1 (BIK1) [77,78]. Mitogen-activated protein kinase (MAPK) cascades are also activated in this process, resulting in the transcriptional activation of PAMP-related genes [79]. ROS production, occurring during pathogen-triggered RBOH activation, plays a role in setting off cellular oxidative conditions that govern plant pathogen responses. This can lead to hormonal signaling, metabolic reprograming, protein redox-PTMs, or cell death related to a hypersensitive response (HR) [74,80]. In *Nicotiana benthamiana*, pathogen-derived signals induced a dramatic increase in protein *S*-sulfenylation, which was attenuated by the silencing of *RBOHB* [74]. This strongly supports the idea that RBOH has an important role in the control over *S*-sulfenylation in response to pathogen stress. The study further demonstrated that pathogen signals induced HR in leaves; this process was sensitive to dimedone. Since, as stated earlier, dimedone reacts with *S*-sulfenylated residues, it was concluded that signaling via *S*-sulfenylation is key for the HR in response to pathogens.

MAPK cascades mediate signal transduction in response to pathogens and a variety of other stresses in plants. Several MAPKs are activated as a result of an increase in cellular or extracellular H_2_O_2_ or other oxidative conditions [75]. In most cases, however, the underlying mechanistic details remain to be established. Arabidopsis proteomic surveys, aiming to identify in vivo targets of *S*-sulfenylation, revealed that several MAPKs, in particular MAPK4, are modified in response to H_2_O_2_ [57,72]. Following the exposure of the Arabidopsis cell culture to H_2_O_2,_ MAPK4 was shown to be modified at Cys^181^, which resides in the Common Docking (CD) motif, a signature MAPK protein interaction domain (Figure 1C) [57]. Using a site-directed mutagenesis approach, Cys^181^ was demonstrated to be critical for in vitr*o* maximal kinase activity [57]. Further analyses were conducted using transgenic plants transformed with MAPK4 variants, where Cys^181^ was replaced by Ser (oxidation-insensitive) or Asp (putative *S*-sulfenylation-mimic) variants [81]. This study demonstrated that the mutation of Cys^181^ into Asp disrupted the proper function of the CD domain and that Cys^181^ is essential for adequate signal transduction in vivo. MAPK4 is involved in mediating plant responses to cold, salt, and pathogens, as well as to cell division [82,83,84]. In particular, MAPK4 is a known suppressor of resistance to *Pseudomonas syringae* [82]. A transgenic plant carrying the Cys^181^-to-Asp variant phenocopied a *mapk4* insertion line, while the one carrying the Cys^181^-to-Ser variant behaved as the WT [81]. The study also further documented the importance of MAPK4 Cys^181^ in plant growth and development. The studies discussed above offer evidence that *S*-sulfenylation is an important mode of signal transduction in plants and the approaches used in these works, including the use of *S*-sulfenylation-mimic variants, offer potent tools with which to study the in vivo relevance of this redox PTM. Nevertheless, while significant progress has been made, the component(s) involved in the reduction of Cys^181^ remain to be identified.

## 3. *S*-Sulfinylation and *S*-Sulfonylation

**Figure 2:**
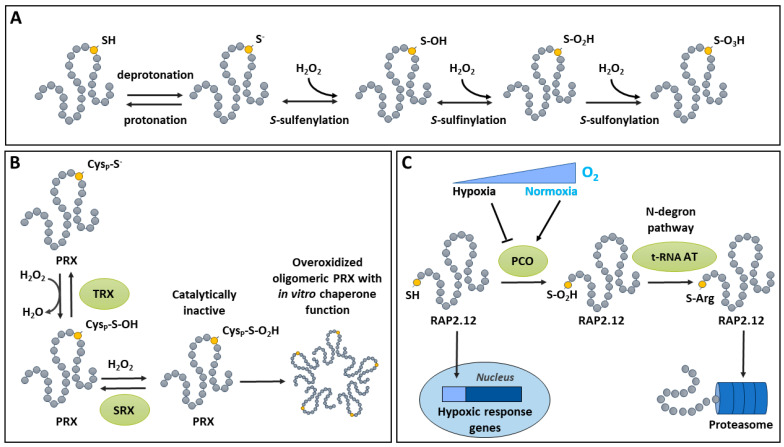
Protein *S*-sulfinylation and *S*-sulfonylation in plants. (**A**): The transition of Cys residues from thiol to *S*-sulfonylated state. When fully protonated (left), Cys residues (yellow dots) are in the thiol state (SH). Depending on conditions prevailing in the environment, the thiol can deprotonate to form a nucleophilic thiolate (S^−^) sensitive to oxidation by H_2_O_2_ treatment. Sequential oxidations of the thiolate by H_2_O_2_ (left to right) lead to its *S*-sulfenylation (SOH), *S*-sulfinylation (SO_2_H), and *S*-sulfonylation (SO_3_H). The last step is considered irreversible. (**B**): The modulation of peroxiredoxin functions by the oxidation status. Peroxiredoxin (PRX) detoxifies H_2_O_2_ to H_2_O using peroxidatic (catalytic) Cys (Cys_P_). The reaction oxidizes Cys_P_ to its sulfenic acid form, which can be reduced back with the help of thioredoxin (TRX). In the presence of H_2_O_2_, the sulfenylated Cys_P_ can be further oxidized to a sulfinic acid. This step is reversible in a reaction catalyzed by sulfiredoxin (SRX). Sulfinylated PRX can lead to the formation of an oligomeric, catalytically inactive PRX displaying in vitro chaperone functions. (**C**): The involvement of plant cysteine oxidases in O_2_ status signal transduction. The transcription factor RAP2.12 positively regulates plant gene expression in response to hypoxia. Under normoxic conditions, RAP2.12 has low stability because it is oxidized by a plant cysteine oxidase (PCO), leading to sulfinylation of N-terminal Cys, which is then arginylated by a tRNA-ARGINYL-TRANSFERASE (t-RNA AT). This process leads RAP2.12 to be degraded by the proteasome. Under hypoxia conditions, PCO is inactivated. This leads to the stabilization of RAP2.12, allowing the promotion of hypoxic-responsive gene expression. See the text for additional details.

Sustained oxidative conditions can lead to the spontaneous overoxidation of sulfenic acid to sulfinic and sulfonic acid forms, which are typically associated with oxidative distress (Figure 2A). The production of sulfonic acid, which is the most highly oxidized species of thiol, is completely irreversible. Sulfinate’s level of oxidation is generally irreversible, except in the specific case of PRXs. The latter are thiol-dependent enzymes that decompose peroxides using a peroxidatic (catalytic) Cys (Cys_P_) (Figure 2B) [53]. During the catalytic cycle of a PRX, its Cys_P_ becomes oxidized to the sulfenic acid form, which is normally reduced using a resolving Cys (Cys_R_) [85]. This leads to the formation of an S-S between Cys_P_ and Cys_R_, which is further reduced by thioredoxin (TRX) to complete the catalytic cycle [85,86]. Interestingly, under oxidative conditions, sulfenic Cys_P_ can become further oxidized to a sulfinic acid [53,85]. Such overoxidized Cys_P_ is catalytically inactive, but can be reduced by an ATP-dependent SRX [87,88], which returns the Cys_P_ to its sulfenic form [53,85]. However, upon overoxidation, plant PRX oligomerizes and acquires a novel function as a chaperone (Figure 2B) [86,89], as documented in other systems [90]. This chaperone activity in overoxidized, high-molecular-weight PRX has been documented in vitro when using insulin and citrate synthase as substrates [91,92]. The physiological relevance of PRX overoxidation and oligomerization has been studied. In most physiological stress condition, there is little change in PRX status, whereas treatment with the herbicide methyl viologen, an efficient ROS inducer [93], can effectively increase PRX’s molecular weight [94]. In addition, PRX can be modified by other PTMs in ways besides overoxidation [53,95]. Thus, further research will be needed to understand the interplay between PRX PTMs, stress conditions, and PRX functions.

In addition to the above-described non-enzymatic oxidation of sulfenic acid, plant cysteine oxidases (PCOs) can catalyze the addition of two oxygen atoms to a thiol group to form a sulfinic acid [96]. PCOs can be classified into two groups [97]. Group A PCOs are ubiquitous in plants and are not regulated by O_2_ tension, whereas the PCOs of Group B are specific to spermatophytes and induced by O_2_ deficiency [97]. In Arabidopsis, a family of five PCOs (AtPCO1 to AtPCO5) has been described, with a high affinity for Cys residues localized at the N-termini of proteins [96]. While a systematic identification of PCO targets needs to be conducted, recent progress has been made in the resolution of the structure of PCOs and the elucidation of their catalytic mechanisms [98,99]. So far, the evidence suggests that PCOs from Group A (e.g., AtPCO4 and AtPCO5) and Group B (e.g., AtPCO2) have significant similarities in terms of the structure of their catalytic sites, with a cupin-like double-stranded β-helix containing a triad of His residues that coordinate metal cofactors [98,99]. PCOs are involved in the oxidation of the N-terminal Cys residues of specific proteins, leading to N-degron pathway-dependent proteolysis. The oxidation of N-terminal Cys to the sulfinic acid form can lead to protein destabilization and degradation through the N-end rule pathway, which is conserved in mammals, bacteria, and plants [100]. Indeed, the overoxidation of N-terminal Cys is required for its arginylation, which then induces proteosomal degradation [100,101]. The role of Cys oxidation in the N-end rule pathway in plants was recently reviewed [102].

The implication of Cys oxidation via PCOs in the signal transduction of oxygen deficiency is supported by studies on RAP2.12, a member of group VII ETHYLENE RESPONSE FACTORs (ERF-VIIs) (Figure 2C). ERF-VIIs are important transcription factors that promote the response to low O_2_ stress (hypoxia) [103]. They contain a highly conserved Cys residue at the N-terminal [104]. In Arabidopsis, when O_2_ becomes limiting for the maintenance of aerobic metabolism, AtPCO1 and AtPCO2 become less efficient for the oxidation of RAP2.12. The kinetic properties of AtPCOs make them less active under the physiological conditions prevailing in the hypoxic cell (pH, O_2_ concentrations), making them excellent candidates to act as plant O_2_ sensors [105]. Thus, under normoxic conditions, PCOs are active and oxidize the N-terminal Cys residue of ERF-VIIs, thereby promoting their arginylation by tRNA-ARGINYL-TRANSFERASE and their degradation by the proteasome [98,106,107]. Low O_2_ availability lowers PCO activity, resulting in the increased stability of ERF-VIIs, which can then fulfill their function as transcriptional activators of the hypoxic response [107]. Furthermore, it was recently demonstrated that this PCO/ERF-VII pathway is under the control of the metabolic energy sensor ‘Target Of Rapamycin’ through a mechanism that allows the coordination of ERF-VII-mediated responses to hypoxia with the energy status of the cell [108].

Oxygen sensing and signaling, achieved via PCO and the Cys/Arg branch of the N-degron pathway, also play important roles in plant development [109]. Indeed, due to the lack of internal O_2_ transport, plant tissues display diffusion-dependent O_2_ gradients and some tissues, such as the shoot apical meristem (SAM), are normally in hypoxic state [109]. This situation may allow O_2_ concentration-dependent stabilization of transcription factors in different cell types and, thus, the regulation of plant development [110], as documented in the case of LITTLE ZIPPER 2 (ZPR2) [109]. ZPR2 has a conserved Cys at position 2 and is a substrate of PCO after the removal of the N-terminal Met by a Met-aminopeptidase [109]. ZPR2 functions as an activator of leaf initiation. It acts by regulating class-III homeodomain-leucine zippers (HD-ZIP III), which are necessary to initiate new primordia from SAM. Thus, hypoxic conditions prevailing in the SAM stabilize ZPR2 and regulate SAM activity [109].

Another important plant process regulated by the overoxidation of Cys and the N-end rule pathway is vernalization. Recombinant AtPCO1-5 can catalyze VERNALIZATION 2 (VRN2) oxidation to sulfinic acid in its N-terminal Cys^2^ residue in vitro [111], leading to its destabilization and degradation. VRN2 is a major regulator of vernalization in Arabidopsis [112]. It is constitutively expressed and stays enclosed in the meristems in aerobic conditions and warm temperature [111]. Low temperatures reduce O*_2_* diffusion and therefore its availability for both respiration and the enzymatic activity of PCOs. The cold also inhibits PCO activity and VRN2 oxidation [111]. These conditions lead to VRN2 stabilization and its subsequent accumulation in plant organs in response to hypoxia and long-term cold exposure [111]. PCOs therefore contribute to stress response signaling during hypoxia and cold exposure. Hence, the regulation of transcription factor stability by catalyzed overoxidation provides an example of how, despite being usually associated with oxidative distress, *S*-sulfinylation can play essential roles in oxidative eustress.

## 4. Disulfide Bridge Formation

**Figure 3:**
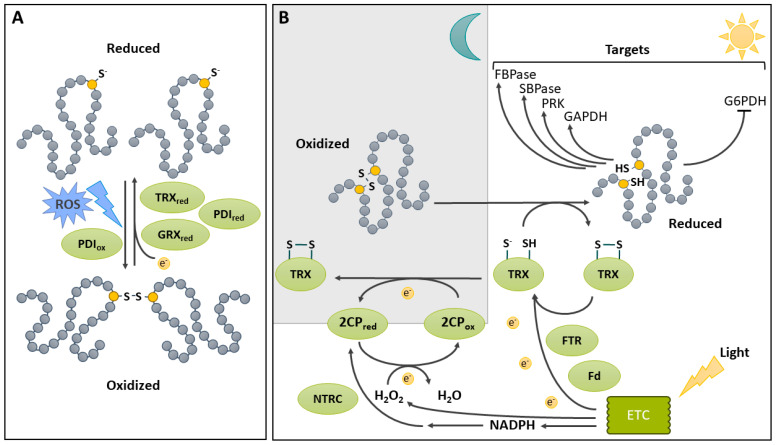
Reversible disulfide bridge formation in plants. (**A**): The reversible formation of disulfide bridge (S-S). Oxidative conditions promoted by ROS and oxidized protein disulfide isomerase (PDI_ox_) in the endoplasmic reticulum favor S-S formation between two adjacent Cys iterations (yellow dots). Thioredoxin (TRX) and glutaredoxins (GRX) reduce S-S concomitantly with the consumption of reducing power (e^−^). In the ER, reduced PDI (PDI_red_) can reduce S-S on misfolded proteins. (**B**): The importance of disulfide bridge formation for metabolic regulation in the light and the dark. In the light (white background), the photosynthetic electron transport chain (ETC) generates reducing power for the reduction of thioredoxins (TRXs) via ferredoxin (Fd) and ferredoxin thioredoxin reductase (FTR). Reduced TRX reduces disulfide bridges in targets in the chloroplast stroma. Activated targets (pointed arrow) include enzymes of the Calvin–Benson–Bassham (CBB) cycle: fructose-1,6-bisphosphatase (FBPase); sedoheptulose-1,7-bisphosphatase (SBPase); phosphoribulokinase (PRK); and glyceraldehyde-3-phosphate dehydrogenase (GAPDH). The oxidative pentose phosphate pathway enzyme glucose-6-phosphate dehydrogenase (G6PDH) is inactivated (blunted arrow). At the onset of dark conditions (gray background), targets become oxidized (S-S). In these conditions, reduced TRX becomes inactive by transferring electrons to 2-Cys peroxiredoxin (2CP), thereby terminating the activation of CBB cycle enzymes. Note that, in the light, 2CP detoxifies H_2_O_2_ generated at the level of ETC and is maintained reduced by NADP-thioredoxin reductase C (NTRC) using photosynthetic NADPH. Subscript red and ox, respectively, symbolize reduced and oxidized states. See the text for additional details.

Two protein Cys residues can form a covalent bond called S-S (Figure 3). The importance of S-S as a key element in protein structure and function has long been recognized. A number of kinetically distinct pathways involving inter- or intramolecular reactions can lead to their generation [113]. These include (i) two-electron Cys oxidation; (ii) one-electron Cys oxidation, involving the formation of a thiyl radical; and (iii) thiol disulfide exchange with a preformed S-S. In order for such covalent bonding to occur, the two intervening Cys residues must come into close proximity (within a few nm) [114]. Once formed, the S-S is a stable covalent bond between two sulfur atoms. Interestingly, the distance between the sulfur atoms is significantly lower for stable structural disulfides compared to reversible, usually regulatory ones (respectively, 2.05 Å vs. 2.18 Å) [115]. The generation of the S-S can be facilitated by the thiolation of one of the Cys residues [28,113]. It can also be promoted by the *S*-glutathionylation of one Cys, which can promote S-S formation with an adjacent Cys [116] or the *S*-nitrosation of an adjacent Cys (see *S*-glutathionylation and *S*-nitrosation sections below). As well, an oxidative environment, such as the conditions prevailing in the lumen of the eukaryotic endoplasmic reticulum (ER), favors S-S formation and proper protein folding [113,117]. The pathways and mechanisms of oxidative protein folding have recently been thoroughly reviewed for plants [118]. In the ER lumen, the oxidative protein folding of nascent proteins is catalyzed by protein disulfide isomerase (PDI). This process consists of a disulfide relay system in which PDI is first oxidized by an oxidized ER oxidoreductin (ERO) [119]. ERO uses O_2_ as an electron acceptor, producing H_2_O_2_ in the process. Reduced PDI can also catalyze the reduction of S-S in misfolded proteins (Figure 3A) [120].

S-Ss notably contribute to maintaining protein structure and stability [15], but also serve vital regulatory purposes [121]. An important aspect of the latter function is the dynamic and reversible nature of S-S formation. Disulfide stability in proteins varies depending on a few parameters, one of which is the dihedral angle of the bond [28]. This feature is influenced by the tertiary structure of the protein. The most stable angle is found at 90°, and there is increasing strain on bonding as the angle diverges from this value [28]. However, the most important impact on disulfides in proteins is due to redox mechanisms responsible for S-S formation via oxidation and their elimination via reduction (Figure 3A). Since the early works on plant enzyme redox modifications, it has been recognized that S-S reduction is mainly catalyzed by thioredoxins (TRXs) [122]. The Arabidopsis genome contains 41 TRX genes [123]. The plant TRX system and its role in metabolism and signaling has been a major area of research and the subject of several extensive reviews in recent years; therefore, the reader is directed to these resources for a more complete overview of the topic [124,125,126,127]. In addition to TRXs, there is now evidence that GRXs can reduce protein S-S [128]. Several reaction mechanisms using GSH as a reductant, have been proposed [128].

### 4.1. Disulfide Bridge Reduction Is an Important Regulatory Mechanism That Links Light Harvesting and CO_2_ Fixation in the Chloroplast

The redox regulation of metabolic enzymes by reversible S-S generation provides sensing of environmental conditions and is especially important in the chloroplast stroma (Figure 3B). This compartment contains the assimilatory enzymes of the Calvin–Benson–Bassham (CBB) cycle, as well as enzymes of the glycolysis and the pentose phosphate pathway (PPP), which use the products of photosynthesis. Plants thus need strict control over these enzymes in order to quickly tune CO_2_ fixation to changes in excitation pressure and to the light/dark cycle [129,130]. Four CBB cycle enzymes are redox-regulated through reversible S-S formation: phosphoribulokinase (PRK) [131,132], heterotetrameric GAPDH [133], fructose-1,6-bisphosphatase (FBPase) [134], and sedoheptulose-1,7-bisphosphatase [135]. In all cases, enzyme activity is inhibited by S-S formation in dark conditions. In addition, homotetrameric GAPDH and PRK are also regulated by interactions with the redox-sensitive CP12 scaffold protein [136]. In this instance, S-S formation on CP12 serves to initiate an interaction between GAPDH, bound to NAD^+^, and CP12; this results in a small decrease in GAPDH activity [137]. The formation of a ternary PRK/GAPDH/CP12 complex is then possible with oxidized PRK, resulting in a much larger decrease in activity for both enzymes [137]. Upon illumination, the chloroplast electron transport chain provides electrons for the subsequent reduction of ferredoxin (Fd), used by Fd-dependent TRX reductase (FTR), to reduce TRX [126,138]. Through a disulfide exchange mechanism, the TRX system mediates the reduction of S-S on target enzymes [126]. A comparative study of the reduction of different targets by the FTR/TRX system suggests that the final electron transfer from TRX to the target enzyme is a rate-limiting step in this redox regulatory process [139]. As noted above, CBB-cycle enzymes are activated by S-S reduction. In contrast, the first enzyme in the PPP, plastidic glucose-6-phosphate dehydrogenase, is activated upon the formation of an S-S between Cys^149^ and Cys^157^ [140]. This oxidation promotes a change in conformation that improves enzyme efficiency. Conversely, the reduction of S-S mediated by reduced TRX *f*1 deactivates the enzyme.

### 4.2. Disulfide Bridge Formation in the Chloroplast under Dark Conditions

As illustrated above, the reduction of chloroplasts enzymes upon illumination is relatively well characterized. However, oxidation mechanisms upon a switch to dark conditions remain poorly understood. Nonetheless, recent studies are providing information that help in the identification of players responsible for the oxidation of reduced targets in the dark (Figure 3B). As seen above, chloroplast redox regulation is highly dependent on TRXs, which must be reduced to catalyze target disulfide reduction in the light. In chloroplasts, there is a second redox pathway, which uses NADPH-dependent TRX reductase C (NTRC). NTRC is implicated in the antioxidant capacity of chloroplasts by reducing 2-Cys peroxiredoxins (2CPs), which are oxidized while scavenging H_2_O_2_ [141]. Additionally, 2CPs can be reduced, although less efficiently, by another plastidic TRX [142]. In the Arabidopsis *ntrc* mutant, the reduction of 2CPs is therefore mediated by the Fd/FTR/TRX system, which causes a depletion in reduced TRX, indirectly affecting the regulation of TRX targets [142]. Decreasing the level of 2CPs in the *ntrc* mutant background enabled the recovery of the WT phenotype, indicating the important role of NTRC in chloroplast redox homeostasis, i.e., regulating 2CPs [142]. Furthermore, a study using genetically encoded redox probes provided further support for the key role of 2CPs in the oxidative inhibition of CBB cycle function [143]. Thus, the Fd/FTR/TRX system for the regulation of CBB cycle enzymes and NTRC/2CPs for H_2_O_2_ detoxification are linked by the redox status of 2CPs [142]. By draining electrons from TRXs, 2CPs allow the fast oxidation of TRXs in the dark, enabling the inactivation of CBC enzymes within 15 min of darkness [144].

## 5. *S*-Glutathionylation

Glutathione synthesis and degradation in plants were recently extensively reviewed [47]. Briefly, the Glu–Cys ligase (GSH1) conjugates the γ-carboxyl group of Glu and the amino group of Cys. Glutathione synthase (GSH2) then uses the resulting γ-glutamylcysteine and Gly to produce glutathione. GSH2 activity is present in the cytosol and the plastid, whereas the step catalyzed by GSH1 is solely localized in the chloroplast and redox-regulated [47]. Thus, glutathione synthesis is linked to the plastid redox state. This sensitivity to redox is mediated by the formation of intramolecular S-S between Cys^178^ and Cys^398^, which activates Arabidopsis GSH1 and has been proposed to act as a redox switch for glutathione synthesis [145].

Glutathione is usually present in mM concentrations in plants, mostly in its monomeric reduced form (GSH) [47]. Oxidative stress promotes the accumulation of its dimeric oxidized form (GSSG). GSSG can be recycled to its reduced form by glutathione reductase (GR), using the reducing power of NADPH [47]. GSH is involved in the cellular redox buffer and the provision of electrons to the Foyer–Halliwell–Asada cycle during H_2_O_2_ detoxification [48]. The value of the GSH/GSSG ratio is therefore linked to the removal of H_2_O_2._ In absence of stress, this ratio is normally very high [48]. The maintenance of an appropriate GSH/GSSG ratio is dependent on GRs. Indeed, the lack of this activity in the cytosol or organellar compartments leads to the accumulation of GSSG, which can be documented using genetically encoded redox sensors [146,147].

**Figure 4:**
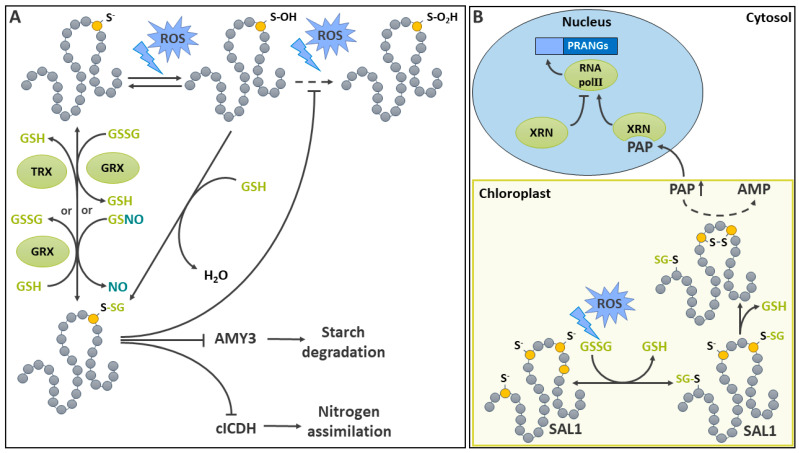
Protein *S*-glutathionylation in plants. (**A**): Mechanisms of *S*-glutathionylation/deglutathionylation and regulation by *S*-glutathionylation. *S*-glutathionylation (S-SG) occurs on deprotonated (S^−^) Cys (yellow dot) in a glutaredoxin (GRX)-catalyzed reaction with oxidized glutathione (GSSG), or spontaneously in a reaction involving nitrosoglutathione (GSNO). It can also occur spontaneously between sulfenylated Cys (S-OH) and reduced glutathione (GSH). Deglutathionylation is catalyzed by GSH-dependent GRX or by thioredoxin (TRX). The *S*-glutahionylation of a Cys residue protects the protein from ROS-dependent oxidation towards the *S*-sulfinylated form (-SO_2_H, top of the panel). *S*-glutathionylation protects α-amylase 3 (AMY3) from overoxidation and inhibits its activity towards starch degradation. AMY3 activity is recovered after stress. It also has an inhibitory effect on cytosolic NADP-dependent isocitrate dehydrogenase (cICDH), which is active in nitrogen assimilation. (**B**): The involvement of the *S*-glutathionylation of SAL1 in expression of plastid redox-associated nuclear genes. SAL1 is a 3′-phosphoadenosine 5′-phosphate (PAP) phosphatase that controls the degradation of PAP to AMP in the chloroplast. In oxidative stress conditions, the *S*-glutathionylation of SAL1 induces the formation of an intramolecular disulfide bridge (S-S) which participates in increasing PAP levels. According to a model, PAP is exported to the nucleus, where it binds to a 5′–3′ exoribonuclease (XRN) involved in the inhibition of RNA polymerase II (RNA polII) transcription of plastid redox-associated nuclear genes (*PRANG*s). PAP binding to XRN relieves the inhibition of RNA polII, allowing *PRANG* transcription. See the text for additional details.

A low cellular GSH/GSSG ratio promotes Cys *S*-glutathionylation [48,51]. This formation of a mixed S-S between glutathione and an accessible protein Cys residue can occur spontaneously [6]. However, with a p*K*_a_ of 8.8, GSH is highly protonated and thus weakly reactive in the physiological pH range, especially towards thiols [148]. Thus, the *S*-glutathionylation reaction (Figure 4A) can involve GSH and a sulfenic acid, or result from a disulfide exchange between GSSG and a thiolate residue [6,148,149]. Nitrosoglutathione (GSNO) has also been shown to act as a mediator of protein *S*-glutathionylation [150]. When tested as an *S*-glutathionylation agent, GSNO was differently effective on various targets [150]. In animals, *S*-glutathionylation appears to be at least partially catalyzed. A study on human glyoxalase II revealed that this enzyme could mediate the in vitro *S*-glutathionylation of specific targets [151]. The involvement of glyoxalase II in *S*-glutathionylation has not been explored in plants so far. In addition, animal glutathione *S*-transferase Pi (GST Pi) also promotes protein *S*-glutathionylation in vivo and in vitro [152]. In contrast to animals, plants lack GST Pi [153]. There is nevertheless a study that has documented the catalysis of *S*-glutathionylation in plants. In this research, plant GRXC2 stimulated the *S*-glutathionylation of the Leu-rich receptor Ser/Thr protein kinase BAK1, using GSSG as substrate [154]. By this means, GRXC2 inhibited BAK1 kinase activity [154]. This mechanism could potentially allow the redox regulation of the brassinosteroid signaling pathway, in which BAK1 is active [154,155]. However, the in vivo significance of BAK1 *S*-glutathionylation remains to be established. There are several examples of the regulatory role of protein *S*-glutathionylation in glycolytic and respiratory metabolism, as reviewed recently [6].

*S*-glutathionylation is fully reversible (Figure 4A). In vitro, strong reductants such as DTT are commonly used to induce non-enzymatic protein deglutathionylation [66,156]. In vivo, reducing conditions such as a high GSH/GSSG ratio promote the removal of glutathione (deglutathionylation) [157]. A study on human PDIs showed a limited capacity for deglutathionylation in vitro [158]; however, this has not been explored in plants. Some evidence for protein deglutathionylation by cyanide has also been provided in mammalian cells [159]. However, so far, this possibility does not appear to have been reported in plants. Deglutathionylation is most likely catalyzed by GRXs in vivo [48,160,161]. Plant genomes encode large GRX gene families, ranging from approximatively 30 genes in Arabidopsis, *Oryza sativa* (rice), and *Populus trichocarpa* (poplar) to 85 in *Triticum aestivum* L. (wheat) [162,163]. Two catalytic mechanisms have been described for the removal of the glutathione moiety on proteins by GRXs [160]. The reduction of the mixed S-S between glutathione and a protein first involves a nucleophilic attack of the modified Cys by a thiolated GRX Cys active site. In the monothiol mechanism, the resulting *S*-glutathionylated GRX is subsequently reduced by GSH, generating GSSG in the process. In the dithiol mechanism, a second Cys attacks the mixed S-S between the GRX and the glutathione, resulting in the formation of an S-S between the two Cys of the GRX and the liberation of GSH. The reduction of the S-S on the GRX later allows it to become active in a new catalytic cycle. In addition to GRXs, TRXs have also been implicated in the deglutathionylation of plant proteins in vitro [66,161]. TRXs and GRXs are related proteins involved in thiol–disulfide exchange [164]. Their substrate specificity is considered to be broad, and they may exhibit some limited overlap. The precise determinants of GRX and TRX substrate specificity remain poorly understood, and will need to be better characterized in the future. Nevertheless, a recent modeling study has shown that electrostatic complementarity could play an important role in determining interactions between the different redoxin isoforms and their interaction partners [165]. Studies conducted in vitro on two Arabidopsis cytosolic GAPDH isoforms show that TRX can catalyze the deglutathionylation of GAPDH in a GSH-independent manner, although less efficiently than GRX [66].

### 5.1. S-Glutathionylation as a Means of Protecting Metabolic Enzymes against Irreversible Oxidation

Because of its reversibility, and the fact that deglutathionylation restores an intact thiol, *S*-glutathionylation has long been recognized as a means of protecting protein Cys against the irreversible oxidation of thiols due to *S*-sulfonylation (Figure 4A) [149,166]. This protective function has been documented for plant metabolic enzymes. Between a few tens and a few hundreds of proteins have been identified as *S*-glutathionylation targets in various plant proteomic surveys [167,168,169,170,171]. Among these, metabolic enzymes are usually abundantly represented. An example of *S*-glutathionylation serving as a protective mechanism against irreversible oxidation comes from a study of ascorbate peroxidases (APXs) in the red alga *Galdieria partita* and in *Nicotiana tabacum* (tobacco) [172]. APX is responsible for H_2_O_2_ detoxification in the Foyer–Halliwell–Asada cycle, but may become inactive in absence of ascorbate due to irreversibly oxidized Cys residues [172]. The *S*-glutathionylation of several APX Cys residues was demonstrated in vitro in the presence of H_2_O_2_ and GSH, and it was suggested that *S*-glutathionylation has a protective role under oxidative stress conditions in vivo [172]. *S*-glutathionylation was also shown to protect Arabidopsis chloroplastic α-amylase 3 (AMY3) activity from overoxidation in vitro [161]. It is thought that this mechanism could allow the recovery of AMY3 function (stress-induced starch degradation) after exposure to oxidative conditions generated under stress [161]. Excess H_2_O_2_ can also cause the irreversible inactivation of GAPDH, a key glycolytic enzyme which is also involved in signaling. This enzyme possesses a catalytic Cys that is highly sensitive to inactivation by H_2_O_2_. In this case also, *S*-glutathionylation was shown to mitigate the effects of oxidative distress in vitro [66].

### 5.2. Metabolic Enzymes Targeted by Regulatory S-Glutathionylation under Oxidative Conditions

There are numerous examples of *S*-glutathionylated enzymes in plant primary metabolism. As seen above, there are multiple cases of redox regulation in chloroplast metabolism. Enzyme sensitivity to reducing power in the organelle is also mediated by *S*-glutathionylation [51]. An analysis of the conservation of *S*-glutathionylated sites in chloroplast proteins provided evidence for the evolutionary conservation of some target proteins [171]. This indicates an ancient origin for the implication of *S*-glutathionylation in chloroplastic stress response. As an example, the *S*-glutathionylation of at least three chloroplastic AMY3 Cys residues has been described in Arabidopsis (Figure 4A) [161]. Among these, Cys^499^ and Cys^587^ were previously shown to be involved in a regulatory S-S, reversible by TRX, in a process similar to redox-modified CBB cycle enzymes [173]. In a proposed model, *S*-glutathionylation of one Cys in the pair led to the formation of the S-S, resulting in the spontaneous deglutathionylation of the other [161]. AMY3 deglutathionylation and S-S reduction were, respectively, promoted by GRX and TRX [161].

The implication of reversible *S*-glutathionylation in the regulation of glycolytic and respiratory metabolism was reviewed a short while ago [6]. More recently, the cytosolic NADP-dependent isocitrate dehydrogenase (cICDH) was shown to be subject to regulatory *S*-glutathionylation (Figure 4A) in a study that provided an example of GSNO as an *S*-glutathionylation agent [174]. By generating 2-oxoglutarate, used as a carbon skeleton in N assimilation, cICDH plays a key function at the interface between C and N metabolisms [175]. The sensitivity of cICDH was demonstrated by decreases in extractable cICDH activity in leaves of the Arabidopsis mutants impacted by H_2_O_2_ detoxification or GSSG reduction [174]. Furthermore, in vitro cICDH activity was inhibited in the presence of GSSG and GSNO, or after treatment with H_2_O_2_ plus GSH [174]. In these assays, GSNO appeared to be particularly effective. Detailed analyses revealed that GSNO induced the *S*-glutathionylation of cICDH on Cys^363^. Following treatments with GSNO, there was also evidence for the *S*-nitrosation of the protein, although the targeted Cys residue(s) could not be identified. ICDH activity could be restored by GRXC1 and GRXC2 and, less efficiently, by TRXs [174].

### 5.3. Involvement of S-Glutathionylation in Signaling

3′-phosphoadenosine 5′-phosphate (PAP) is a product of sulfotransferases [176,177]. In some instances, PAP has been described as a potent retro-inhibitor of these enzymes [178]. It is also a product and an inhibitor of the stromal acyl carrier protein synthase [179]. PAP also partakes in retrograde signaling between the chloroplast and the nucleus, as it regulates plastid redox-associated nuclear genes (*PRANG*s) [180]. PAP is degraded to AMP by the chloroplastic PAP phosphatase SAL1 [181,182]. Under normal conditions, low levels of PAP are therefore controlled by SAL1 activity in a process that involves *S*-glutathionylation (Figure 4B). Under stress-induced oxidative conditions, Arabidopsis SAL1 activity decreases in conjunction with its dimerization, its *S*-glutathionylation, and the formation of an intramolecular S-S between evolutionarily conserved Cys^167^ and Cys^190^ [183]. Treatment of SAL1 with GSSG in vitro promoted *S*-glutathionylation of Cys^119^ and Cys^190^ and down-regulated monomeric and dimeric SAL1 activity. These experiments also revealed the existence of a mechanism by which a prior Cys *S*-glutathionylation induced the formation of a S-S between Cys^167^ and Cys^190^ by means of a thiol disulfide exchange, leading to the downregulation of SAL1 [183]. This inhibition of SAL1 leads in turn to an increased steady state level of PAP, which acts as a chloroplast-to-nucleus retrograde signal [181]. Later studies have led to the development of a model where PAP accumulation allows it to bind to and inhibit 5′–3′ exoribonucleases involved in *PRANG*s expression by the degradation of uncapped RNAs, interference with RNA polymerase II function, and/or silencing [182].

## 6. Protein Persulfidation

**Figure 5:**
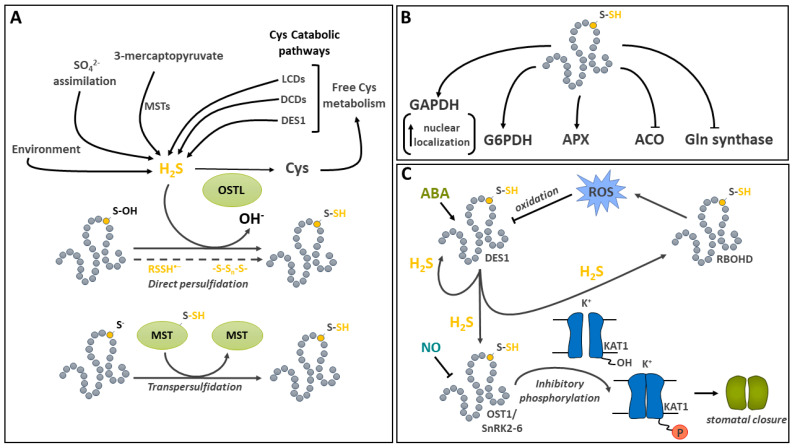
Protein persulfidation in plants. (**A**): Pathways controlling cellular H_2_S together with the mechanisms of persulfidation. In the top of the panel, pathways that promote cellular H_2_S levels are represented. These include uptake from the environment, SO_4_^2−^ assimilation, 3-mercaptopyruvate sulfurtransferases (MSTs), and the catabolism of free Cys by L-Cys desulfhydrases (LCDs), D-Cys desulfhydrases (DCDs), and the cytosolic *O*-acetylserine (thiol)lyase (OSTL) homolog DES1. OSTL consumes H_2_S for Cys synthesis. Note that H_2_S is used, although HS^−^ is probably present in physiological conditions. The bottom of the panel represents the two pathways implicated in protein persulfidation. The direct persulfidation pathway modifies Cys residues (yellow dots) in the *S*-sulfenylated form (S-SH) using free H_2_S. Other mechanisms of direct persulfidation (dotted arrow) do not have strong experimental support. The transpersulfidation pathway allows the transfer of a persulfide group from MST to a thiolated Cys (S^−^). (**B**): Metabolic targets of persulfidation in plants. The effect of persulfidation is represented (pointed arrow: increased activity; blunt arrow: inhibition). Some target names are abbreviated: ACO, aminocyclopropane-carboxylic acid oxidase; APX: cytosolic ascorbate peroxidase; G6PDH, glucose-6-phosphate dehydrogenase; GAPDH: cytosolic glyceraldehyde 3P dehydrogenase. Increased nuclear localization has also been reported as a result of GAPDH persulfidation. (**C**): The involvement of persulfidation in signal transduction. H_2_S serves to activate DES1 by persulfidation. Upon abscisic acid (ABA) stimulation, DES1-derived H_2_S modifies Open Stomata 1/Sucrose nonfermenting 1-RELATED PROTEIN KINASE2.6 (OST1/SnRK2-6), which is inhibited by NO signaling and responsible for the inhibitory phosphorylation of the KAT1 potassium channel in the guard-cell membrane. The phosphorylation of KAT1 inhibits potassium transport, leading to stomatal closure. In a regulatory negative feedback loop, the modification of Respiratory Burst Oxidase Homolog D (RBOHD) by H_2_S activates the process, leading to ROS formation and the inhibition of DES1. See the text for additional details.

Hydrogen sulfide (H_2_S) is an important intermediate in the plant sulfate assimilatory pathway [184] as well as a gaseous pollutant that can be absorbed by plants [184,185,186]. It has recently emerged as a signaling molecule in plants [35,187,188]. The modification of a protein thiol by H_2_S is called persulfidation. In the literature, it is also sometimes referred to as protein persulfuration or protein sulfhydration. However, the latter terminology is considered incorrect since no hydration reaction is involved [54]. This PTM allows H_2_S-based signal transduction. The production of H_2_S occurs in several subcellular compartments. During sulfate assimilation, it is generated in plastids by sulfite reductase [184] and transferred to *O*-acetylserine (OAS) by OAS(thiol)lyases (OSTLs) to generate Cys (Figure 5A) [184,189,190]. Apart from the sulfur assimilation pathway, the production of intracellular H_2_S by several other plant pathways has been recently reviewed [189,190]. These mainly involve the catabolism of Cys by L-Cys desulfhydrases (LCDs), D-Cys desulfhydrases (DCDs), and the cytosolic OSTL homolog DES1 (Figure 5A). LCDs and DCDs produce H_2_S, ammonia, and pyruvate in the cytosol, while DES1 breaks down Cys into H_2_S and OAS. Other enzymes are involved, such as the mitochondrial β-cyanoalanine synthase (CAS), which catalyzes the conversion of Cys and hydrogen cyanide (HCN) into H_2_S and β-cyanoalanine. In addition, a small family of 3-mercaptopyruvate sulfurtransferases (MSTs) characterized in Arabidopsis [191] also generate H_2_S, using 3-mercaptopyruvate as sulfur donor and reduced TRX or GRX as electron donors (Figure 5A). Furthermore, upon reaction with reduced Cys or GSH, they, respectively, generate Cys persulfide (Cys-SSH) and glutathione persulfide (GSSH) [191].

### 6.1. Addition of Sulfide on Cys Results from Direct Persulfidation or Transpersulfidation

Several mechanisms can lead to the spontaneous persulfidation of Cys (Figure 5A). In aqueous solutions, H_2_S dissociates in hydrosulfide (HS^−^, p*K*_a_ = 7.0 at 25°) and sulfide anions (S^2−^, p*K*_a_ = 17–19 at 25 °C) [54]. Thus, even if we use the term H_2_S in this review, the more nucleophilic HS^−^ is probably the most abundant form at physiological pH values [54]. HS^−^ can perform a nucleophilic attack on an oxidized thiol, such as with a sulfenic acid or a disulfide, but cannot react with a reduced thiol [192]. Other means of protein persulfidation have been discussed [54], such as those involving radical sulfur species (RSSH^•–^) or inorganic polysulfide (-S-S_n_-S-); however, the relevance of these reactions in plants still remains to be clearly established. Experimentally, p-methoxyphenyl(morpholino)phosphinodithioic acid (GYY4137) and NaHS are used as sulfide donors for protein modification and physiological studies [185,193,194,195].

In Arabidopsis, the ability of MSTs to catalyze a transpersulfidation reaction (transfer of a sulfide from one protein to another, Figure 5A) has been demonstrated in vitro using roGFP2 as a model protein substrate [191]. In this reaction, MST becomes persulfidated on its catalytic Cys following interactions with its substrate 3-mercaptopyruvate. This occurs in the absence of TRX or GRX. The transfer of sulfide occurs from MST to a thiolate residue on roGFP2. Following a rearrangement, the persulfidated Cys forms a disulfide bridge with a nearby thiol, resulting in the generation of H_2_S [191]. Interestingly, the catalytic Cys of MST is also subject to inhibitory oxidation by H_2_O_2_. The persulfidation of this residue also has a protective role against the irreversible oxidation of the MST [191].

Cys modification by persulfidation is reversible in vitro with artificial reducing agents such as DTT and tris(2-carboxyethyl)phosphine (TCEP) [194,196]. In animals, redoxins can reduce protein persulfides and their levels are controlled by the thioredoxin system [54,197]. It is quite possible that this process also takes place in plants; however, this remains to be formally demonstrated.

### 6.2. Metabolic Targets of Cys Persulfidation

Over the past decade, high-throughput proteomic methods have been developed and used to survey the extent of protein persulfidation in plants [198,199,200,201]. Based on a biotin switch method, a large number of persulfidated proteins have been identified in Arabidopsis, showing the widespread occurrence of this PTM in leaves and roots as well as its regulation by environmental conditions [199,200]. The comparison of various proteomic studies revealed that persulfidation seems far more abundant in Arabidopsis than *S*-nitrosylation or *S*-glutathionylation [202].

Persulfidation has been implicated in the regulation of key enzymes in the metabolic pathways such as Gln synthetase in N assimilation, G6PDH in the OPPP, GAPDH in glycolysis, cytosolic ascorbate peroxidase (APX) in the ascorbate–glutathione cycle, and aminocyclopropane-carboxylic acid oxidase (ACO) in ethylene synthesis (Figure 5B) [194,203]. In a study of APX, in vitro activity was shown to be modestly stimulated by persulfidation, whereas Gln synthetase was inhibited by treatment with NaHS at nM concentrations [194]. For ACO, the incubation of recombinant proteins with NaHS led to reversible inhibition by the persulfidation of Cys^60^, and treatments of *Solanum lycopersicum* (tomato) plants with the H_2_S donor further supported the inhibition of in vivo enzyme activity by persulfidation [203]. In a recent study on Arabidopsis and tomato G6PDHs, persulfidation was shown to play an important role in the regulation of the activity of these enzymes, which catalyze C entry in the OPPP [204]. G6PDH modification was detected on Cys^155^ in Arabidopsis G6PDH6 and Cys^159^ in tomato G6PDHC. The same residues were also shown to be sensitive to oxidation by H_2_O_2_. G6PDHs are structurally relatively well conserved between plants and animals. The fact that the persulfide-modified residues are only found in cytosolic isoforms suggests plant- and isoform-specific modification [204]. In vitro and in vivo treatments with NaHS, inducing the persulfidation of G6PDH6 and G6PDHC, enhanced enzyme activity. This effect was reversed in the presence of DTT. Further analyses indicated that G6PDH persulfidation increased the affinity of NADP, used as a substrate, and promoted enzyme oligomerization towards the formation of tetramers. In addition, the exposure of Arabidopsis seedlings to salt stress caused the oxidation of Cys^155^ and resulted in decreased enzyme activity. However, in the presence of NaSH, competition between oxidation and persulfidation occurred, highlighting the potential of Cys^155^ persulfidation in the protection of G6PDH activity under oxidative conditions, although mechanistic details remain to be clarified.

The persulfidation of Arabidopsis cytosolic GAPDH C1 was shown to reversibly increase enzyme activity in vitro [194]. Proteomic surveys have shown that cytosolic and chloroplastic GAPDH isoforms are modified in vivo by persulfidation [199]. The relative localization to the nucleus and the cytoplasm of GAPDH isoforms C1 and C2 was compared in WT and the *des1* mutant of Arabidopsis [205]. Decreased localization to the nucleus was reported in the *des1* background, whereas treatment of the mutant with NaHS increased nuclear localization. These results support the hypothesis of preferential nuclear localization upon GAPDH persulfidation [205]. Moonlighting functions and nuclear localization have been reported before for animal and plant GAPDHs under various stress conditions [206]. In Arabidopsis, the persulfidation of GAPDH appears likely to promote its migration to the nucleus. However, its nuclear function still remains to be deciphered [205,206].

### 6.3. Cys Persulfidation Involvement in ABA-Mediated Stomatal Movement

A wide variety of signaling functions have been shown to be impacted by persulfidation in plants, including in abiotic stress tolerance [35,188,207]. In particular, it has been implicated in drought stress signaling by regulating abscisic acid- (ABA-) mediated stomatal movement (Figure 5C) [208,209,210]. The ABA regulation of guard-cell function implicates a complex network of signals, comprising protein kinases and H_2_O_2_-, NO-, and H_2_S-regulated steps [208,211]. Initial investigations showed that, in the guard cell, ABA induces the production of ROS, which is linked to the activity of NADPH oxidases RBOHD and F [212]. The synthetic H_2_S donor GYY4137 was then shown to inhibit the activity of the *Nicotiana tabacum* inward-rectifying K+ channel [193]. Among the protein kinases involved in ABA signal transduction, Open Stomata 1/Sucrose nonfermenting 1-RELATED PROTEIN KINASE2.6 (OST1/SnRK2.6) is responsible for mediating the phosphorylation of Thr^306^ on the inward K^+^ channel KAT1, thereby reducing K^+^ uptake by the guard cell and promoting stomatal closure in Arabidopsis [213]. Recently, it was demonstrated that ABA signaling induces the persulfidation of Cys^44^ and Cys^205^ on DES1, a major source of H_2_S in the cytosol [209]. This PTM leads to the enhancement of DES1 activity in an autoactivating mechanism. The production of H_2_S by DES1 leads to the persulfidation of RBOHD on Cys^825^ and Cys^890^, thereby stimulating their activity and promoting the production of ROS [209]. In turn, the oxidation of the persulfidated Cys residues on DES1 due to the rise of ROS provides a negative feedback mechanism, leading to a decrease in DES1 activity [209]. DES1 mediated H_2_S production in guard cells also contributes to the mediation of ABA signaling by promoting the persulfidation of OST1/SnRK2.6 on Cys^131^ and Cys^137^ [208]. Interestingly, the persulfidation of this key protein kinase increases its activity [208], while *S*-nitrosylation on Cys^137^, which is close to the catalytic site, is inhibitory [214]. This complex cross-talk between H_2_S and NO signals, acting as second messengers in various aspects of plant physiology, has been recently reviewed [215]. So far, there is strong evidence that, in guard cells, the two molecules collaborate in the fine regulation of components of ABA signaling [187].

## 7. *S*-Cyanylation

**Figure 6:**
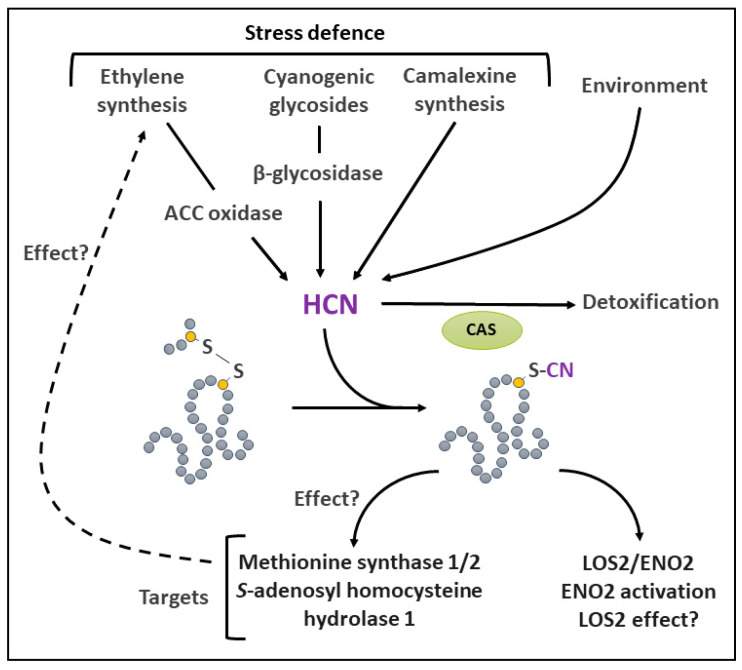
Protein *S*-cyanylation in plants. The main pathways involved in the generation of HCN are represented. HCN can also be taken from the environment. The reaction of β-cyanoalanine synthase (CAS) consumes HCN in plants. *S*-cyanylation (purple symbols) occurs mainly on Cys residues (yellow dots) engaged in disulfide bonds. Among the few targets of *S*-cyanylation identified in plants, the glycolytic enzyme Enolase 2 (ENO2) was shown to be activated via modification. Other targets were identified, and some were involved in pathways leading to ethylene synthesis. As indicated by question marks, many effects of *S*-cyanylation are still largely unknown. See text for additional details.

*S*-cyanylation is a PTM resulting from the reaction of HCN on a protein Cys residue (Figure 6). HCN is a pollutant naturally present at low levels in the environment, where its presence is often the result of human activity [216]. It is volatile and can dissociate into H^+^ and CN^−^ when dissolved in aqueous solutions (p*K*_a_ = 9.2). Therefore, in the physiological pH range, HCN mainly occurs in its undissociated form. HCN can be formed enzymatically or non-enzymatically in a variety of living systems, from bacteria to mammals [217,218,219]. It is widely recognized as a poisonous compound due to its enzyme inhibitory effects, the most important of which act on cytochrome c oxidase [220], although its physiological function as a gasotransmitter is also currently debated [219].

In plants, a number of pathways, many of which relate to stress defense, are implicated in the generation of HCN (Figure 6). The latter is produced during the synthesis of ethylene by 1-aminocyclopropane-1-carboxylate (ACC) oxidase [221]. Therefore, biotic and abiotic stresses, as well as developmental situations that promote ethylene synthesis [222], result in HCN formation. The hydrolysis of cyanogenic glycosides by β-glycosidase is another source of HCN in a relatively large number of plant families [217,220]. This so-called cyanogenesis mechanism is thought to be an effective deterrence strategy upon wounding or attack by herbivores [220]. HCN is also formed during the synthesis of the phytoalexin camalexin [223] and in a reaction that uses glyoxylate and hydroxylamine [224]. However, the enzymatic mechanism responsible for this reaction remains elusive. Plant cellular HCN can be detoxified by CAS [225,226]. However, this reaction also produces HS^−^, which, at high concentrations, can inhibit cytochrome c oxidase [227]. In addition, as seen above, HS^−^ is also a protein Cys modifier.

In animal and plant systems, HCN has been reported to form protein adducts in vivo by *S*-cyanylation [228,229]. HCN’s nucleophilic properties allow a non-enzymatic attack of S-Ss, including those present in proteins [219,230,231]. As a result, the bond is broken and *S*-cyanylation most likely occurs on the Cys residue which is the farthest from an electrophilic group [230,232]. It is thought that HCN can also attack the mixed disulfide bridge between GSH and Cys, resulting in the deglutathionylation of the Cys. in vitro, protein *S*-cyanylation is promoted by the use of an oxidative treatment (e.g., H_2_O_2_), presumed to induce S-S formation [229]. Although the CN adduct can be eliminated in vitro [233], *S*-cyanylation is regarded as an irreversible PTM in living systems [229].

There is still limited information available on the occurrence and the physiological relevance of protein *S*-cyanylation in plants. Nevertheless, in a ground-breaking study, the feeding of an Arabidopsis *cas*-null mutant with ACC was used to increase endogenous HCN levels in order to detect *S*-cyanylated proteins [229]. This strategy allowed the identification of 163 targets. Among the modified proteins, there was an enrichment of metabolic enzymes involved in non-photosynthetic and photosynthetic carbon metabolism. One of the identified targets was Enolase 2 (ENO2), which catalyzes the penultimate step of the cytosolic glycolytic pathway. ENO2 was shown to be activated by *S*-cyanylation on Cys^346^, hinting to the possible involvement of HCN in the regulation of glycolytic flux (Figure 6). Interestingly, the locus *LOS2*/*ENO2*, which encodes for ENO2, also produces a truncated form of the protein, C-MYC BINDING PROTEIN1 (LOS2), which serves as a transcriptional regulator. The effect of HCN on this protein is however unknown. Among the other *S*-cyanylation targets identified in this study were Met synthase 1 and 2, as well as *S*-adenosyl-homocysteine hydrolase 1 (Figure 6). These enzymes are involved in Met and *S*-adenosyl Met metabolism. Their modification by *S*-cyanylation could therefore impact methylation reactions, gene silencing, or ethylene synthesis [229]. However, this still remains to be established. More recently, another proteomic study using the *cas*-null mutant confirmed that enzymes involved in Met and *S*-adenosyl Met metabolism are the preferred targets of *S*-cyanylation in plant roots [234].

Obviously, much remains to be performed to further understand the physiological relevance of these findings. This will require a careful examination of the function of *S*-cyanylation targets. In addition, future research efforts will need to consider the fact that *S*-cyanylation only affects oxidized Cys residues (involved in S-S or mixed disulfide bonds).

## 8. *S*-Nitrosation

**Figure 7:**
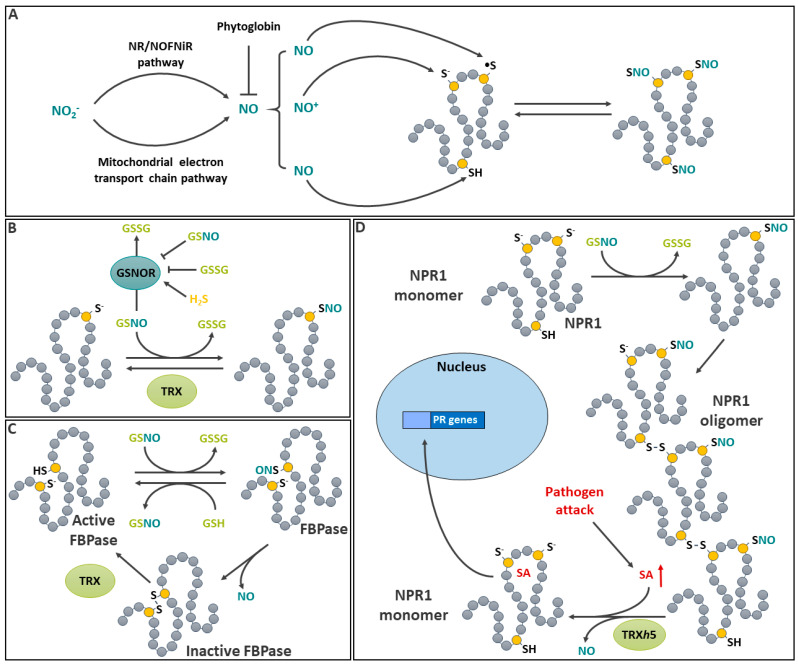
Protein *S*-nitrosation in plants. (**A**): The generation of NO in plants and the modification of Cys by direct *S*-nitrosation. NO can be formed from NO_2_^−^ by a pathway that involves nitrate reductase (NR) and NO-forming nitrite reductase (NOFNiR). It can also be formed by reduction of NO_2_^−^ by the mitochondrial electron transport chain. NO can be scavenged by phytoglobin. The direct reactions of NO with Cys (yellow dots) thiolate (S^−^), thyil (^●^S) or thiol (SH) can lead to *S*-nitrosation (SNO). (**B**): Protein transnitrosation by *S*-nitrosoglutahione. *S*-nitrosoglutahione (GSNO) is a stable form of NO with can be used in the transnitrosation of proteins. GSNO is catabolized by GSNO reductase (GSNOR), which is susceptible to inhibition by GSNO and oxidized glutathione (GSSG) and stimulated by H_2_S. (**C**): The regulation of fructose-1,6-bisphosphatase (FBPase) by reversible *S*-nitrosation. In its reduced form (SH, top left), FBPase is active. High GSNO levels promote the *S*-nitrosation of FBPase (top right), whereas high glutathione (GSH) levels revert it. The *S*-nitrosated FBPase can undergo the formation of a disulfide bridge (S-S, bottom) that inhibits FBPase activity. S-S reduction by thioredoxin (TRX) occurs in the light and restores FBPase activity. (**D**): The regulation of NONEXPRESSOR OF PATHOGENESIS-RELATED GENES1 (NPR1) by *S*-nitrosation. NPR1 is a receptor for the phytohormone salicylic acid (SA), which transcriptionally regulates systemic acquired resistance through the induction of *Pathogenesis-Related* (*PR*) genes. In the absence of pathogen stress, NPR1 is *S*-nitrosated by GSNO and sequestered in the cytosol in the form of large oligomers with an inter-subunit S-S. Upon pathogen attack, TRX*h*5 promotes the monomerization of NPR1 by reducing inter-subunit S-S and catalyzing its denitrosation. In turn, reduced monomeric NPR1 can enter the nucleus, where it promotes *PR* gene transcription. See the text for additional details.

Nitric oxide (NO) is a gaseous free radical that can easily diffuse across membranes. In plants, NO synthesis can be achieved through several pathways, most of which involve the reduction of nitrite [235]. Several details in plant NO production are not yet fully understood. Although sequences with homology to animal nitric oxide synthase (NOS) have been found in plants, the role of NOS in plants NO synthesis remains elusive [235]. It appears that metabolic routes involving the reduction of NO_2_^−^ are more likely to contribute to the production of plant NO (Figure 7A). A reaction involving nitrate reductase (NR), which catalyzes the reduction of NO_3_^−^ to NO_2_^−^, has been implicated in the process [235]. To produce NO, electrons from NR are supplied to NO-forming nitrite reductase (NOFNiR), leading to the reduction of NO_2_^−^ to NO [235]. Hence, *NR* mutants (e.g., *nia1* and *nia2* in Arabidopsis) are often used to decrease NO production in planta [236]. There is another pathway, in which the reduction of NO_2_^−^ to NO by the mitochondrial electron transport chain under anaerobic conditions has been documented [237]. Following its formation, NO can be scavenged by plant hemoglobins (Phytoglobins, Pgbs), which are induced by O_2_ deficiency. The evidence for this was initially provided by studies in transgenic *Medicago sativa* (alfalfa) roots [238] and *Zea mays* (maize) cell cultures [239] with different *Pgb* expression levels. Under O_2_ deficiency conditions, NO levels were inversely correlated to Pgb expression level in both cases.

### 8.1. Mechanisms Involved in Protein S-Nitrosation

NO can react directly with protein Cys to produce a PTM called *S*-nitrosation. This denomination is used here to describe the modification of a Cys residue by NO instead of the commonly used *S*-nitrosylation, as the latter refers to the modification of a metal by NO [240]. NO plays an important role in plants, animals, and bacteria as a second messenger and controls a wide variety of biological functions [241]. Direct protein *S*-nitrosation (Figure 7A) occurs via different reaction mechanisms of NO with protein Cys and has been reviewed in some detail [240,242]. These reactions are complex and probably facilitated by additional redox reactions occurring under physiological conditions [242]. Briefly, direct *S*-nitrosation can be the result of (i) a thiolate reaction with the nitrosonium cation (NO^+^) formed by NO oxidation by transition metals, (ii) the reaction of NO with a protein thyil radical, or (iii) a Cys thiol reaction with NO, in which the participation of a second NO molecule leads to the production of a nitrosated Cys and HNO [243]. A transnitrosation reaction can also lead to *S*-nitrosation [241]. The latter can be associated with the denitrosation of the NO donor [244]. Although all these mechanisms promote protein *S*-nitrosation, it is important to note that *S*-nitrosation, induced by different reactions (e.g., radical reaction and transnitrosation), does not necessarily have the same protein target specificity [241,245]. However, this issue is understudied in plants.

In plant tissues, GSNO acts as a stable NO reservoir for spontaneous protein *S*-nitrosation [246]. GSNO is recognized as the main NO donor for transnitrosation reactions (Figure 7B), although some enzymes, such as human GAPDH, may also possess trans-nitrosase activity [247]. Mechanisms affecting cellular GSNO will therefore indirectly impact *S*-nitrosation. The spontaneous reaction of the glutathione thiyl radical with NO has been described as a pathway leading to the formation of GSNO [247]. In plants, it is assumed that the main enzyme regulating levels of GSNO is *S*-nitrosoglutathione reductase (GSNOR), a cytosolic class III alcohol dehydrogenase implicated in GSNO catabolism [246,248,249]. GSNOR catalyzes the NADH-dependent reduction of GSNO to GSSG and NH_3_ [248]. This enzyme has three conserved solvent-accessible Cys that are sensitive to redox modifications. A study on *Lotus japonicus* revealed that LjGSNOR1 and LjGSNOR2 can be modestly activated by persulfidation and inhibited by *S*-glutathionylation [250]. However, the latter reaction was slow (several h) and required mM concentrations of GSSG. It is thus unclear if the *S*-glutathionylation of GSNOR is relevant in vivo. Arabidopsis GSNOR is also inhibited by H_2_O_2_ in vitro and by conditions promoting oxidative stress in vivo [251]. Interestingly, tomato GSNOR is reversibly inhibited by the *S*-nitrosation of solvent-accessible Cys^271^ [252,253]. This inhibition has been proposed to be involved in a mechanism that would allow proper NO signaling during nitrosative burst by allowing initial GSNO accumulation [252]. More recently, enzymes belonging to the aldo-keto reductase (AKR) family were identified as NADPH-dependent GSNO reductases in mammals [254] and Arabidopsis [255]. Two Arabidopsis AKR4C are significantly upregulated in a *GSNOR* null mutant, which displays a higher NADPH-dependent GSNO reduction rate than the WT, suggesting that AKR4Cs are involved in GSNO homeostasis and compensate for the loss of GSNOR [255].

### 8.2. Protein Denitrosation

The current knowledge on plant denitrosation was recently extensively reviewed [256]. Briefly, protein denitrosation can involve enzymatic or non-enzymatic reactions (Figure 7B–D). In Arabidopsis, cytosolic TRX*h*5 can catalyze the denitrosation of specific proteins [257]. The implication of other TRXs in plant protein denitrosation remains to be tested; however, the high amino acid sequence similarity between TRX isoforms suggest that it is likely [256,257]. Despite their sequence similarities, TRX isoforms differ in their subcellular localization and specific interactions, which could influence their substrate specificity [256]. PRX IIE is involved in a trans-denitrosation mechanism for the bZIP67 transcription factor [244]. Protein denitrosation activity was documented for animal SRX [258] and GRX [259], but the relevance of these enzymes in plant protein denitrosation remains to be investigated. Some enzymes, such as GAPDH [260] and GSNOR [261], can be denitrosated in an enzyme-independent way by direct reaction between GSH and the *S*-nitrosated thiol, resulting in GSNO production (see example below).

### 8.3. Targets of Protein S-Nitrosation in Plant Metabolism

Plant protein *S*-nitrosation has now been studied for over 20 years using proteomic surveys employing the biotin switch technique [262,263,264]. Several hundred *S*-nitrosated proteins, together with *S*-nitrosation sites, have been identified. Among these, the Arabidopsis GAPC1 isoform of cytosolic GAPDH is known to be strongly inhibited by GSNO-mediated *S*-nitrosation on its catalytic Cys^149^ [260]. GAPC1 denitrosation is promoted in vitro by GSH, but not by the TRX system [260]. Furthermore, the GSH-dependent denitrosation is influenced by the GSH/GSNO ratio, but unaffected by the GSH/GSSG ratio [260]. These results contrast with the mechanism taking place in animal GAPDH, which is effectively denitrosated by TRX and resistant to GSH-mediated denitrosation [265,266]. Although *S*-nitrosation is currently more studied than denitrosation, the latter certainly deserves to be more thoroughly investigated, as there appears to be some protein- and/or organism-specific mechanisms at work.

*S*-nitrosation can also be involved in cooperative Cys modification for metabolic regulation. This remarkable mechanism has been documented with *Pisum sativum* (pea) FBPase. This CBB cycle enzyme can be *S*-nitrosated by GSNO in light conditions (Figure 7C), when the enzyme is normally present in its reduced form (see Figure 3B) [267]. When fully reduced, FBPase is active. A high GSNO/GSH ratio promotes the *S*-nitrosation of FBPase Cys^153^. This *S*-nitrosation can be reversed if the GSH concentration increases (i.e., under low GSNO/GSH), leading to the formation of GSNO. If conditions do not favor GSH-dependent denitrosation, the presence of the *S*-nitrosated Cys^153^ induces a rapid S-S between Cys^153^ and adjacent Cys^173^ with the concomitant loss of NO. The newly formed S-S effectively inactivates the enzyme. In the absence of TRX activity, such as in dark conditions, the enzyme is kept in this oxidized and inactive form. Under conditions where light favors TRX reduction, the S-S can then be reduced and the enzyme becomes active [267]. FBPase has long been known to be regulated by reversible S-S formation. The effects of its *S*-nitrosation provide additional sensitivity to environmental factors that may affect the NO status and the chloroplastic GSNO/GSH ratio, allowing fine-tuning of the CBB cycle function.

### 8.4. Involvement of S-Nitrosation in Signal Transduction Pathways

*S*-nitrosation also plays an important role in signal transduction by affecting other major PTMs (e.g., sumoylation, phosphorylation or acetylation) that are involved in biochemical regulation. This topic was recently reviewed [268]. At the physiological level, it is now recognized that *S*-nitrosation plays a role in important aspects of plant hormone signaling [269]. This is, for example, the case for the ABA signaling during stomatal closure. As discussed above, the activity of protein kinase OST1/SnRK2.6, which is involved in the regulation of the KAT1 inward K^+^ channel, is stimulated by persulfidation (Figure 5C). Investigations into the regulation of this protein kinase demonstrated the negative regulation of ABA signaling by NO [214]. In particular, *gsnor1-3*, an Arabidopsis mutant with increased NO accumulation, was insensitive to the closure of stomata induced by ABA. Further investigations demonstrated that OST1/SnRK2.6 activity was inhibited in vitro in the presence of the NO donor GSNO. This inhibition was caused by the *S*-nitrosation of conserved Cys^137^, which was stimulated in vivo as a result of ABA treatment. It is possible that this work has even deeper significance. Indeed, *S*-nitrosation of Cys^137^ could be relevant to the regulation of other evolutionary-related protein kinases [214].

A second example of the importance of *S*-nitrosation in signaling is found in SA signal transduction. In this process, NONEXPRESSOR OF PATHOGENESIS-RELATED GENES1 (NPR1) is regulated by reversible *S*-nitrosation (Figure 7D). *NPR1* is a key regulator of SA signaling and systemic acquired resistance (SAR); it was first identified using a genetic screen that aimed to identify genetic lesions causing a lack of the systemic response normally induced by *Pseudomonas syringae* [270]. SA, which is involved in the positive regulation of SAR [271], binds to NPR1 [272]. Under normal conditions, NPR1 can be found as a high-molecular-weight oligomer with inter-subunit S-S (Figure 7D) [273]. NPR1 is *S*-nitrosated by GSNO on Cys^156^, which promotes its oligomerization and sequestration in the cytoplasm [274]. Upon a pathogen attack, there is an increase in endogenous SA and SA binding to NPR1. Upon SA accumulation, TRX*h*5 catalyzes the conversion of NPR1 oligomers to monomers, which are translocated to the nucleus to activate the expression of pathogenesis-related genes [273]. The monomerization of NPR1 involves TRX*h*5 in two ways: it reduces the inter-subunit S-S [274] and catalyzes its denitrosation [257].

## 9. *S*-Carbonylation by Reactive Carbonyl Species

**Figure 8:**
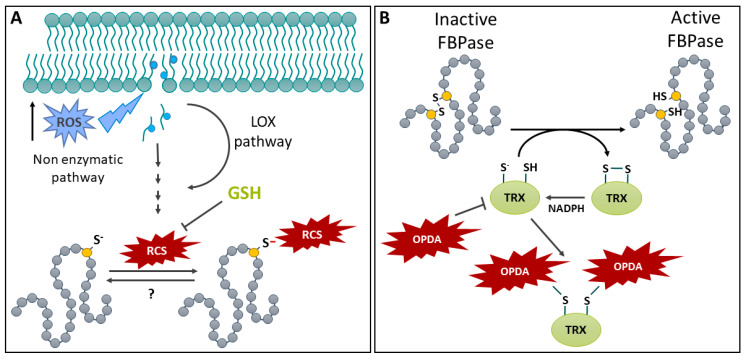
Protein *S*-carbonylation in plants. (**A**): The generation of reactive carbonyl species (RCS) and their reaction with protein Cys residues. Two main pathways are involved in the generation of RCS. The non-enzymatic pathway results from the attack of membrane-derived unsaturated fatty acids (marked with blue dots) by reactive oxygen species. The enzymatic pathway proceeds via lipoxygenase (LOX). Both pathways can produce a variety of reactive electrophilic α,β-unsaturated carbonyl compounds, collectively termed RCS. The detoxification of RCS can occur and is often associated with reduced glutathione (GSH) consumption. RCS can modify Cys residues (yellow dots) in thiolate form (-S^−^), leading to protein *S*-carbonylation. The modification is widely considered irreversible as the decarbonylation reaction, indicated with a question mark, is still hypothetical in plants. (**B**): The regulation of thioredoxin by *S*-carbonylation. The RCS cis-(+)-12-oxophytodienoic acid (OPDA) is formed enzymatically in the LOX pathway. Thioredoxin *f*1 (TRX) is responsible for the reduction of disulfide bridges on fructose-1,6-bisphosphatase (FBPase), thereby activating the enzyme. The in vitro modification of TRX with OPDA (*S*-OPDAylation) renders TRX unable to activate FBPase. See the text for additional details.

Membrane lipids are a major target of oxidation, especially under stress conditions, due to their abundance and proximity to ROS and free radical production sites, such as RBOH and mitochondrial and chloroplastic electron transport chains [41,275,276,277]. This leads to the oxidative degradation of polyunsaturated fatty acids (PUFAs) and subsequent oxylipin production (Figure 8A). Oxylipins are also produced enzymatically, mainly by lipoxygenases (LOXs) that act on linoleic acid and α-linolenic acid (Figure 8A) [275,278]. This enzymatic pathway can lead to the production of important stress signaling molecules such as cis-(+)-12-oxophytodienoic acid (OPDA) and jasmonic acid (JA) [275,278]. Oxylipins also comprise more than a dozen species containing a reactive electrophilic α,β-unsaturated carbonyl moiety; they are collectively termed reactive carbonyl species (RCS) [279]. RCS include, but not exclusively, compounds such as acrolein, OPDA, 4-hydroxy-2-nonenal (HNE), and malondialdehyde (MDA) [279]. In this review, we follow the restrictive definition of RCS given in the review by Mano [279]. Nevertheless, it is important to note that RCS definitions appear to vary between authors and that species such as methylglyoxal or some aldehydes are regularly referred to as RCS, although they are not included in the previous definition [279]. The latter publication also specifies the terms reactive electrophile species and oxylipins, which are sometimes interchanged with RCS in the literature [279].

Since the lipid composition and the abundance of PUFAs vary between subcellular membranes, some, such as the chloroplast, appear to be major RCS production sites [280,281]. Furthermore, the hypothesis of stress- or compartment-specific RCS signatures (usually referred to as oxylipin signature) is increasingly supported by the literature [278,282,283,284]. RCS production, effect, target specificity, and detoxification vary between members of the RCS group and this was recently reviewed [280,285].

In plants, RCS can be detoxified enzymatically. Various enzymes are involved, and they display some specificity towards the structure of their RCS substrate(s). Enzyme activities such as aldehyde dehydrogenase, aldehyde reductase, aldo-keto reductase, 2-alkenal reductase, alkenal/one oxidoreductase, and glutathione transferase Tau (GST τ) are implicated in RCS detoxification [286]. In a survey of the in vitro activity of 23 GST τ isoforms with different RCS, acrolein and HNE were the preferred substrates out of 11 isoforms [286]. Non-enzymatic RCS scavenging also occurs and involves the formation of a conjugate between RCS and polyphenols [287] or GSH. RCS detoxification by the GSH pool (Figure 8A) under conditions of a high GSH/GSSG ratio is illustrated in a study of Arabidopsis overexpressing GR. The overexpression of GR led to higher levels of GSH and a higher GSH/GSSG ratio, which were associated with an enhanced RCS detoxification capacity compared to the WT [288].

### 9.1. Interrelations between RCS and ROS Signaling

RCS are produced downstream of ROS and their production is increased during oxidative stress [280]. Like ROS, RCS can cause both eustress (signaling) and distress (damage) [285,289]. In a study on *Chlamydomonas reinhardtii,* acrolein treatment at a low dose significantly upregulated the genes involved in GSH, S, and ascorbate metabolism, and the redox homeostasis enzymes leading to acclimation to ROS [289]. However, RCS distress, caused by higher doses of acrolein, led to the loss of cell viability [289]. RCS could thus act downstream of ROS as signal in plant stress responses [281,285,290,291,292,293]. However, it remains to be seen whether any of these effects involve Cys carbonylation.

Nevertheless, there is evidence that RCS impacts the redox network. RCS may affect the cellular glutathione contents and redox state, as illustrated with high-acrolein treatments [289,293,294]. As discussed above, RCS enzymatic or non-enzymatic detoxification, consuming GSH, could deplete the GSH pool and increase the GSSG/GSH ratio. This could indirectly impact redox-sensitive Cys PTMs, for instance, by altering protein *S*-glutathionylation [294]. However, a formal demonstration of this effect has yet to be provided.

RCS have a longer half-life than ROS and their shared characteristics with lipids allow them to diffuse through membranes [295]. The greater diversity of RCS compared to ROS could contribute to allowing for a greater specificity of downstream ROS signaling [290]. Thus, RCS should be considered in the pursuit of understanding how stress-specific and compartment-specific signaling pathways are generated [281,296].

### 9.2. Protein Thiols Modification by RCS and S-OPDAylation

RCS electrophilic β carbon allows the covalent modification of targets such as nucleic acids and proteins [281]. Because of its reactivity, Cys is a prominent RCS target, although other protein amino acids (e.g., His, Lys) can also be modified [297,298]. RCS can modify Cys residues via a Michael reaction mechanism, resulting in primary protein carbonylation [278]. The reactivity of the RCS modifier, as well as its availability, is controlled by its formation/scavenging rates, which are thought to control the nature of protein carbonyls [280,281]. Furthermore, protein carbonyls can undergo secondary reactions, leading to irreversible and complex protein modifications [281]. Thus, protein carbonylation is traditionally considered irreversible. However, some reports in animals show some support for thiol-dependent decarbonylation [299,300]. In plants, protein decarbonylation is still poorly characterized. In a report, MDA-induced protein carbonylation was partially reversed, but the mechanism remains elusive [301]. It is possible that the reversibility of Cys carbonylation varies in relation to the function of the target protein and/or the primary and secondary modifications induced by RCS.

The effect of RCS modifications on the specific targets of the redox network in plants was recently reviewed [278] and Cys modifications of specific targets are beginning to be identified [302,303]. Nevertheless, progress in this area is still limited, perhaps due to the complexity of the detection of primary and secondary modifications. The cytotoxic and reactive species acrolein and HNE are among the most studied RCS [277,294]. There are reports that these compounds may cause specific enzyme inhibition in plants [294,304]. So far, no direct evidence links these inhibitions to Cys modification. However, this hypothesis certainly deserves consideration since the enzyme activity of several reported RCS targets [294] is known to be sensitive to Cys oxidation.

OPDA is structurally related to the cyclopentenone prostaglandin hormones found in animals [303]. Prostaglandins are known to exert some of their physiological functions through the oxidation of Cys thiols [305]. In plants, recent studies demonstrated that OPDA can react with protein thiols, resulting in *S*-OPDAylation [303]. In vitro TRX*f*1 modification by OPDA was detected and Cys modification was documented by mass spectrometry. This *S*-OPDAylation led to the inhibition of TRX*f*1 to reductively activate fructose-1,6-bisphosphatase (Figure 8B) [303]. The subsequent inhibitory *S*-OPDAylation of chloroplast TRX*m*4 and cytosolic TRX*h*3 and *h*5 was also detected [306]. The data suggest a unified mode of action for OPDA on TRXs. However, individual *S*-OPDAylated residues were not confirmed by mass spectrometry in the latter study.

ODPA is a precursor of jasmonic acid, a phytohormone implicated in responses to biotic and abiotic stress [307]. Similarly to OPDA, which is reported to modulate key elements in plants redox regulation [306], cyclopentenone prostaglandins target the essential components of the redox regulation network in animal cells under stress [305,308]. Prostaglandin regulatory mechanisms, exerted by protein redox PTM, provide a perspective and a possible working model worth exploring in terms of the action mode of OPDA in plants.

## 10. *S*-Acylation

**Figure 9:**
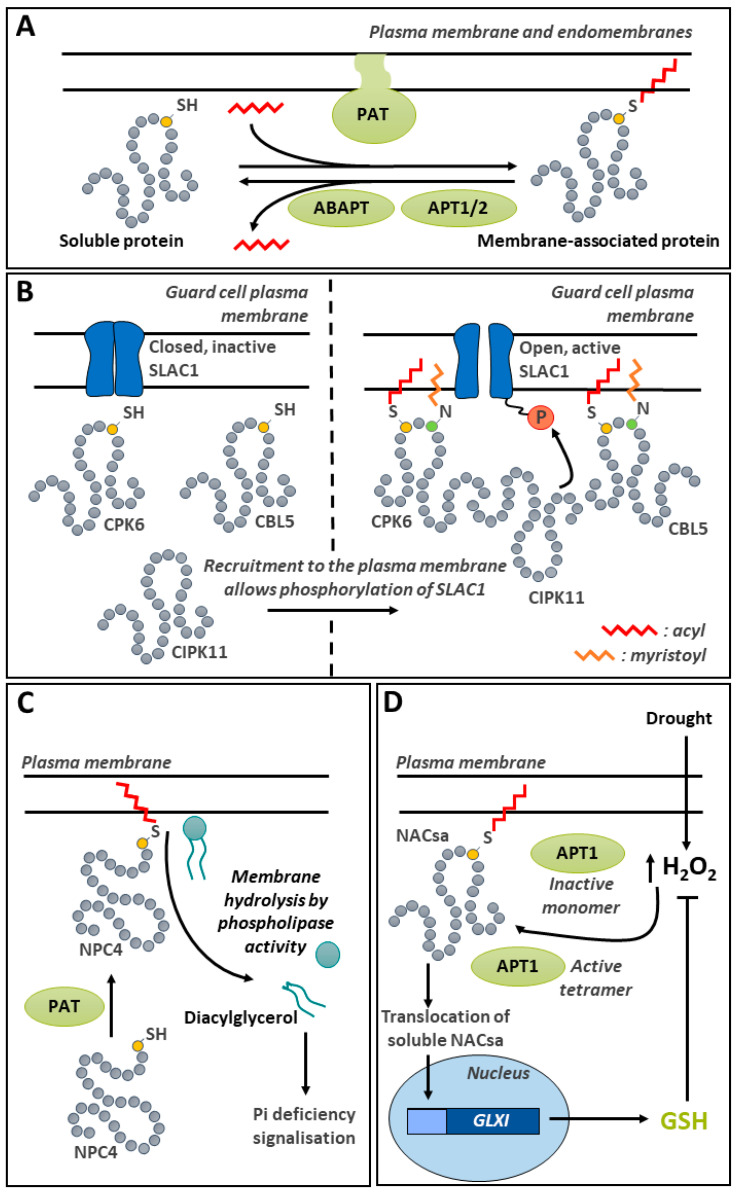
Protein *S*-acylation in plants. (**A**): *S*-acylation and de-*S*-acylation. The membrane-localized protein *S*-acyl transferase (PAT) catalyzes the addition of an acyl group (red jagged line) onto a Cys (yellow dot) thiol present on a soluble protein. The addition allows the *S*-acylated protein to become membrane-associated. The reverse reaction (de-*S*-acylation) is catalyzed by ABHD17-like acyl protein thioesterases (ABAPT) or acyl protein thioesterases (APT), which release the acyl group and solubilize the protein. (**B**): The involvement of *S*-acylation in the regulation of SLAC1. In the guard-cell plasma membrane, the slow anion channel 1 (SLAC1) is a key element in abscisic acid-dependent stomatal closure. The channel is activated by phosphorylation. A double lipid modification (N-myristoylation on a Gly (green dot) and *S*-acylation) allow the membrane localization of otherwise soluble Calcineurin B-like protein 5 (CBL5) and Ca^2+^-regulated protein kinase CPK6. This allows the recruitment of Calcineurin B-like protein-interacting protein kinase 11 (CIPK11) by CBL5 and the phosphorylation (activation) of SLAC1. (**C**): The involvement of *S*-acylation in Pi deficiency signalization. The non-specific phospholipase C4 (NPC4) is *S*-acylated on a C-terminal Cys. Its phospholipase activity allows it to generate diacylglycerol that acts in Pi deficiency signalization. (**D**): The involvement of the de-*S*-acylation of transcription factor NACsa in signal transduction. Under normal conditions, the transcription factor NACsa is sequestered to the plasma membrane by *S*-acylation. In drought conditions, an increase in H_2_O_2_ activates monomeric acyl protein thioesterase 1 (APT1) to take its tetrameric form. Tetrameric APT1 deacylates NACsa and releases it from the membrane, allowing its translocation to the nucleus, where it activates the transcription of *Glyoxalase 1* (*GLX1*). This promotes a glutathione build-up, acting to inhibit H_2_O_2_ accumulation. See the text for additional details.

In plants, the chloroplast is the main site for de novo fatty acid production [309]. *S*-acylation (also known as *S*-palmitoylation) consists of the reversible modification of a Cys residue by a fatty acid, usually palmitic acid (C16) or stearic acid (C18), through a thioester bond [310]. Among the known lipid modifications of proteins, *S*-acylation is unique because of its reversibility, thus making it a dynamic means of regulation [310,311]. *S*-acylation is not a trivial PTM in plants. A recent study in different Arabidopsis tissues identified 1849 Cys residues modified by *S*-acylation on 1094 different proteins using an acyl-resin-assisted capture (acyl-RAC) strategy [312]. The meristem and the silique contained the most acylated proteins. While about half of protein acylation was contributed by a single tissue, this rate was found to be only 6.5% in all tissues studied, indicative of a certain tissue specificity level. *S*-acylation is catalyzed by a family of enzymes called protein *S*-acyl transferases (PATs, also called DHHCs) that contain an Asp-His-His-Cys (DHHC) motif shown to be necessary for activity [313]. This motif is conserved in PATs found in microorganisms, animals, and plants [314,315,316]. PATs are membrane proteins that are distributed in several different cellular membrane compartments (Figure 9A) [317]. The Arabidopsis genome contains 24 PATs, with half of them being targeted to the plasma membrane [317], while the rest localize to different endomembrane systems. This situation contrasts with yeast and mammalian PATs, which are predominantly localized to the ER and Golgi [317]. Another unique feature of *S*-acylation is that it does not occur on conserved sites or consensus sequence motifs [318]. It is rather thought that it is influenced by the ability of the catalytic site of PATs to access their target Cys [319]. It is currently unknown if PATs are redox-regulated, but the possible modification of the conserved Cys in the DHHC motif could have an impact on *S*-acylation activity. Recently, a proteomic survey identified PAT24 as a persulfidation target in Arabidopsis roots [200]. *Pat24* mutants display an altered root hair phenotype, indicating that *S*-acylation, catalyzed by PAT24, plays an important role in root hair growth [320].

### 10.1. S-Acylation Appears to Be Critical for Target Proteins Membrane Localization

The effect of *S*-acylation on proteins that lack transmembrane helices (TMHs) is an increase in their hydrophobicity, thereby promoting their association with membranes (Figure 9A). For example, among all *S*-acylation target proteins identified in Arabidopsis, the vast majority (73%) lacked TMH [312]. Accordingly, this suggests that *S*-acylation may significantly influence the cellular membrane distribution for target proteins. However, many proteins already associated with membranes can also be *S*-acylated [321]. It is thus unlikely that the function of protein *S*-acylation is limited to membrane anchoring. Early studies suggested that *S*-acylation of a Cys residue in close proximity to a transmembrane domain (TMD) may serve to influence the conformation of the TMD within the membrane [322]. However, recent investigations of this effect in plants suggest that a careful validation of the epitope-tag methods used to study these phenomenon is warranted before drawing strong conclusions [310]. Nevertheless, several studies document the impacts of *S*-acylation on the regulation of signal transduction and cell wall metabolism.

The cellulose synthase complex (CSC) is responsible for the synthesis of the most abundant plant polymer. The CSC is a large transmembrane protein complex consisting of at least 18 cellulose synthase (CESA) subunits [323]. It is located at the plasma membrane where it consumes UDP–glucose on the cytoplasmic side and generates nascent cellulose microfibrils on the apoplastic side. Multiple CESA subunits were found to be *S*-acylated [324], with an estimated 100 *S*-acylated Cys per CSC. Out of the twenty-six Cys in Arabidopsis CESA7, four Cys of the Variable Region 2 (VR2) and two others, found at the C-terminal end (CT), were *S*-acylated and were studied using a site-directed mutagenesis approach in transgenic lines. This indicated that VR2 region Cys residues were important in terms of plant cellulose contents [324], and that, while VR2 and CT Cys are not essential for CSC assembly at the endomembrane level up to the Golgi vesicles, they are required for correct localization to the plasma membrane [324]. Thus, the *S*-acylation of the CSC subunits may control cellulose production by allowing the correct intracellular trafficking of the complex to the proper site for cellulose production after its assembly on endomembranes.

The importance of protein *S*-acylation can further be illustrated by its impact on several signaling pathways in plants, as previously reviewed [318]. In recent years, there has been mounting evidence that Ca^2+^ sensing and signaling proteins are *S*-acylated. It was shown that the *S*-acylation of several Ca^2+^ sensor Calcineurin B-like proteins (CBLs) is required for their membrane positioning and their signaling function in ion homeostasis. For example, the proper localization of Arabidopsis CBL6 was shown to depend on the *S*-acylation of several Cys residues at the N-terminus of the protein [325]. This also impacted the correct targeting of CBL6-interacting protein kinases in planta, implying that the modification of CBL6 is responsible for the targeting of the protein complex [325].

In another study, CBL5 and the Ca^2+^-regulated protein kinase CPK6 were shown to be subject to a double lipid modification (N-myristoylation and *S*-acylation), which was required for the proper localization of the two proteins to the plasma membrane (Figure 9B) [326]. The dual modification allowed the recruitment of Calcineurin B-like protein-interacting protein kinase 11 (CIPK11) by CBL5 in a process that is involved in the regulation of guard-cell slow anion channel 1 (SLAC1) (Figure 9B). SLAC1 is a key element in ABA-dependent stomatal closure. Further studies using heterologous expression in Xenopus oocytes indicate that the dual lipid modification of CBL5 is critical for the activation of SLAC1 via phosphorylation by the CBL5/CIPK11 complex [326]. Thus, this example illustrates the impact of N-myristoylation and *S*-acylation on the regulation of stomatal function.

*S*-acylation is also involved in plant responses to phosphate (Pi) deficiency (Figure 9C). Arabidopsis non-specific phospholipase C4 (NPC4) is a promiscuous enzyme that hydrolyzes membrane phospholipids, generating a free head group and diacylglycerol, which is involved in signaling [327]. The importance of NPCs in plant signaling for growth development and stress has recently been reviewed [328]. In NPC4, the *S*-acylation of Cys^533^, located at the C-terminal extremity, was shown to be necessary for its plasma membrane association [329]. Palmitate was identified as the main modifier. Using a mutation analysis strategy, the *S*-acylation of Cys^533^ was shown to be responsible for successfully restoring NPC4 function in an *npc4* mutant [329]. These data strongly support the involvement of a single *S*-acylation site in the proper localization of NPC4, thereby allowing it to access its substrate(s) and fulfill its signaling functions.

### 10.2. De-S-Acylation Players and Evidence of Their Involvement in Plant Signal Transduction

In animals, two distinct types of proteins, acyl protein thioesterases (APT) [330] and ABHD17-family depalmitoylases [331], have been implicated in the de-*S*-acylation of proteins by cleaving the Cys–fatty acid thioester bond. A group of 11 Arabidopsis hydrolases, named ABHD17-like acyl protein thioesterases (ABAPTs), was recently identified. They share a conserved ABHD region with mammalian ABHD17 proteins and were shown to catalyze de-*S*-acylation in plants and act using a similar catalytic mechanism (Figure 9A) [332].

Plants seemingly share poor sequence homology with mammalian APT1 and APT2 [310]. Nonetheless, a few studies support the role of APT1 in plant de-*S*-acylation (Figure 9A,D) [333,334]. In Medicago species, NACsa is a transcription factor localized at the plasma membrane through Cys^26^ *S*-acylation (Figure 9D) [333]. Under drought stress condition, APT1 catalyzes NACsa de-*S*-acylation, thereby promoting its translocation to the nucleus and the transcription of glyoxalase I (GLXI). GLXI activation increases the GSH/GSSG ratio [333]. These data indicate that APT1 is a mediator of cellular redox potential under drought. Interestingly, APT1 is itself redox-regulated. Its sequence contains three redox-sensitive Cys—Cys^20^, Cys^22^ and Cys^23^—that can be *S*-glutathionylated in control conditions [334]. The *S*-glutathionylation of APT1 promotes its inactive, monomeric form. Drought increases intracellular H_2_O_2_, leading to oxidative stress and promoting the formation of a tetrameric APT1 complex with de-*S*-acylase activity. The de-*S*-acylation of NACsa leads to increased GLXI and a higher GSH/GSSG ratio, contributing to the restoration of redox homeostasis and promoting *S*-glutathionylation and the inactivation of APT1 [334]. As a result, APT1 behaves as an effective redox sensor during drought.

Recently, de-*S*-acylation was identified as a key mechanism in hormone cross-talk. Brassinosteroids (BRs) are important plant hormones, mainly involved in plant growth and development [335], while salicylic acid (SA) is associated with plant defense and stress responses [336]. BR signaling involves the interaction of brassinosteroid insensitive 1 (BRI1) receptors and brassinosteroids signaling kinases (BSKs) at the plasma membrane [335]. The binding of BRs to BRI1 leads to its activation and the phosphorylation of BSKs, initiating the BRs signaling cascade [335]. In normal conditions, BSKs are maintained at the plasma membrane by *S*-acylation, allowing their interaction with BRI1 [337]. Stresses, such as pathogen infection, induce an important increase in SA biosynthesis and signaling [336]. SA increases the expression of ABAPT11 [337]. This promotes the de-*S*-acylation of BSKs, reducing their membrane localization and thus impairing their function in BR signalization [337].

## 11. Prenylation

**Figure 10:**
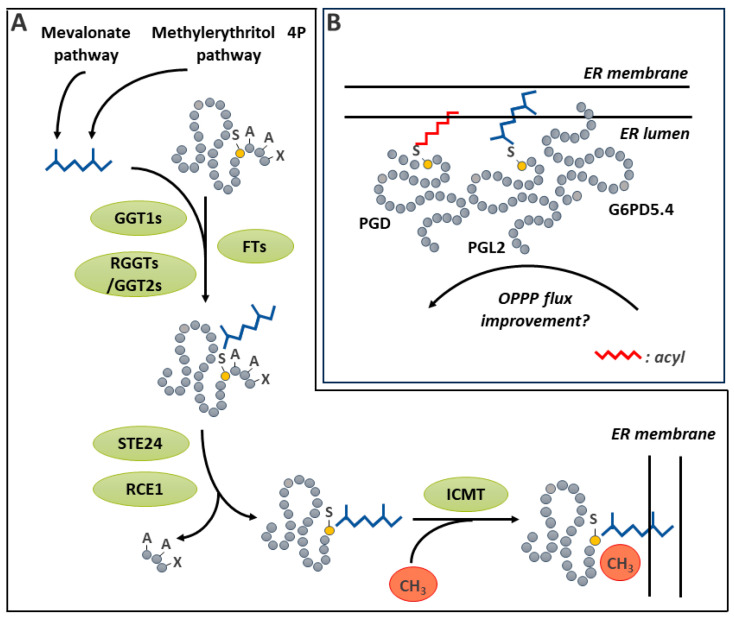
Protein prenylation in plants. (**A**): Pathways responsible for prenyl group synthesis and mechanism of protein prenylation. Prenyl groups (blue colored structure representing isoprenoid C15 or C20 chains) are synthesized by the mevalonate or the methylerythritol 4P pathways. Farnesyl transferases (FTs), geranylgeranyl transferases (GGT-1s), and Rab-geranylgeranyl transferases (RGGTs/GGT-2s) modify the Cys (yellow dot) present in a Cys-A-A-X (A = aliphatic and X = any amino acid) motif located at the C terminus, directing the protein to the ER membrane. The proteolytic removal of C-terminal the AAX sequence is catalyzed by STE24 or RCE1 endo-proteases, and a methylation of the prenylated Cys is performed by a protein-*S*-isoprenylcysteine O-methyltransferase (ICMT). (**B**): The involvement of prenylation in the formation of an oxidative pentose phosphate pathway (OPPP) in the ER. Prenylated 6-phosphogluconolactonase (PGL2) is localized to the ER membrane and interacts with ER *S*-acylated (red jagged line) phosphogluconate dehydrogenase (PGD). PGL2 recruits glucose-6P dehydrogenase 5.4 (G6PD5.4) to the membrane. The result constitutes a metabolon containing the first three steps of the OPPP in ER the lumen. This mode of organization could improve C flux in the OPPP. See the text for additional details.

Protein prenylation has been known to take place in plants for quite some time (see the review by [338]). Protein prenylation is the irreversible covalent modification of a Cys residue by isoprenoids. The known modifiers are 15- (farnesyl moiety) or 20- (geranylgeranyl moiety) carbon chains. These isoprenoids are formed by the condensation of 5-carbon units (isopentenyl pyrophosphate and its isomer dimethylallyl pyrophosphate) that are produced by two plant isoprenoid biosynthetic routes, namely, the mevalonate pathway in the cytoplasm and the methylerythritol 4P pathway in the chloroplast (Figure 10A) [339]. Protein modification by isoprenoids is catalyzed by protein prenyltransferases. These enzymes are classified as farnesyl transferases (FTs), geranylgeranyl transferases (GGT1s), and Rab-geranylgeranyl transferases (RGGTs, also called GGT2s), depending on their specificity for various isoprenoids (Figure 10A) [340]. Protein prenylation is widely considered as a means of anchoring proteins in membranes, in particular in the secretory pathway [341,342]. Prenylation occurs at the C-terminal end of proteins in a consensus site. In the case of FT and GGT1, they modify the consensus Cys-a-a-X motif, where a represents an aliphatic residue and X any amino acid [343]. This prenylation step directs the modified protein to the endoplasmic reticulum (ER), where it is followed by the proteolytic removal of the aaX sequence, which is catalyzed by STE24 or RCE1 endoproteases (Figure 10A) [342]. The resulting free carboxyl residue of the prenylated Cys then becomes available for methylation by a protein-*S*-isoprenylcysteine O-methyltransferase (ICMT) [342]. The subcellular localization of the post-prenylation processing machinery supports ER membrane localization for proteins with the Cys-a-a-X motif; however, they can also follow the secretory pathway [342].

The RGGT/GGT2 class recognizes targets that are bound to the Rab Escort Protein and carry consensus sequences such as Cys-Cys, Cys-X-Cys, Cys-Cys-X, Cys-Cys-X-X, and Cys-Cys-X-X-X, in which both iterations of Cys present in the motif can be modified [344]. Despite the above-described classification of protein prenyltransferases in two categories, distinguished by consensus sequences, protein substrate recognition may not be strictly exclusive [345]. An extensive searchable protein prenylation database that includes plants is available to facilitate research on this topic (https://mendel.imp.ac.at/PrePS/index.html, accessed on 30 July 2024) [346].

Plant protein prenylation has been the subject of numerous reviews, including a recent one on its involvement in plant stress responses [343] and another on its signaling aspects [347]. We chose to only focus on a single recent study exemplifying the impact of prenylation in metabolism. This concerns the organization of the OPPP enzymes (Figure 10B). OPPP commits carbon to the generation of NADPH and carbon skeletons used in redox homeostasis and anabolic pathways [348]. Traditionally, this pathway has been thought to operate in the cytosol and the plastid stroma [348], while some steps have also been found in the peroxisome [349]. In Arabidopsis, one of the isoforms of the second OPPP enzyme (6-phosphogluconolactonase, PGL2) contains a C-terminal Cys-a-a-X motif [350], indicating its possible prenylation. Additionally, all three phosphogluconate dehydrogenases (PGD, the third step of the OPPP) can be *S*-acylated [312,350]. In an intriguing study, the presence of PGL2 at the ER was documented. Moreover, this subcellular localization was shown to be dependent on the Cys residue of the Cys-a-a-X motif. Furthermore, there is evidence that PGL2 recruits G6PD5.4 in specific ER subdomains. G6PD5.4 is a functional isoform of glucose-6P dehydrogenase, arising by the alternative splicing of *G6PD5* and by carrying an N-terminal extension serving as ER membrane anchor. Additional evidence suggests that *S*-acylated PGD2 may interact with the first two OPPP enzymes [350]. Thus, a combination of G6PD5.4 N–terminal ER membrane targeting, together with PGL2 prenylation and PGD2 *S*-acylation, allows the constitution of a metabolon regrouping the first three steps of the OPPP on the cytosolic side of the ER membrane. It is hypothesized that this mode of organization should improve the metabolic flux in the OPPP (Figure 10B). Since the significance of this organization also depends on the proportion of OPPP enzymes involved in the ER metabolon relative to other subcellular localizations, the study by Linnenbrügger [350] opens up exciting future research opportunities.

## 12. CoAlation

Coenzyme A (CoA) is an ubiquitous cofactor in prokaryotes and eukaryotes [351]. Its five steps biosynthetic pathway from panthothenate has been elucidated for Arabidopsis [352,353]. Protein modification by CoA (CoAlation) was first described in animal [354] cells and shortly after in bacteria and Amoebozoa [355,356]. These studies were greatly facilitated by the use of an anti-CoA antibody able to detect protein CoAlation. This PTM can occur spontaneously. It is promoted by oxidative stress conditions in vivo and fully reversible in vitro using DTT [354,355,356]. A large number of CoAlation targets were identified, many of which were involved in metabolic processes [354,355]. Such a widespread and evolutionary ancient occurrence of the protein CoAlation suggests that it could also occur in plants. However, the evidence for this is still extremely limited. A study on soybean protein Tyr phosphatase, performed by mass spectrometry, suggested the possible CoAlation of the recombinant enzyme produced in *E. coli*, in addition to its *S*-glutathionylation [167]. The characterization of this PTM does not appear to have been pursued. It is unknown so far if this finding is only a reflection of the experimental conditions [167].

## 13. Thiohemiacetal Formation

Thiohemiacetal formation is the result of a Cys nucleophilic attack on an acyl group. It is a reversible reaction, for example, being involved in the catalytic mechanism of a number of dehydrogenases [357,358]. In this case, the thiohemiacetal is a reaction intermediate involving a covalent bond between an enzyme’s catalytic Cys and its substrate [358]. In less common instances, however, thiohemiacetal formation may have regulatory functions. For instance, in plants, thiohemiacetal formation is involved in the regulation of betaine aldehyde dehydrogenase (BADH). BADH catalyzes the oxidation of betaine aldehyde (BAL), which constitutes the last step in the biosynthetic pathway of the major plant osmoprotectant glycine betaine (GB) [359]. BADH catalytic Cys^291^ forms a thiohemiacetal as part of its catalytic mechanism. Interestingly, in a structural study of *Spinacia oleracea* (spinach) BADH, it was shown that Cys^450^, a non-catalytic residue, also undergoes thiohemiacetal formation with BAL [360]. This reaction hinders substrate binding, effectively inhibiting BADH activity. This mechanism was discussed as a possible regulatory mechanism that would prevent excessive NAD^+^ consumption during GB synthesis [360].

Studies on plant mitochondrial alternative oxidase (AOX) provide other evidence for the possible involvement of thiohemiacetal formation in regulatory mechanisms. AOX is an inner mitochondrial membrane dimeric terminal oxidase. It diverts electrons from the cytochrome pathway, bypassing complexes III and cytochrome c oxidase and thereby reducing respiratory ATP yield [361]. The reversible formation of an inter-subunit S-S is involved in AOX regulation. AOX carrying the S-S is inactive. When NAD(P)H levels are high in the mitochondrial matrix, there is reduction of the disulfide bridge, leading to AOX with basal activity. Pyruvate, as well as various matrix α-ketoacids such as glyoxylate, oxaloacetate, and α-ketoglutarate, then interact with the reduced thiols and fully activate AOX. The formation of a thiohemiacetal between the reduced Cys residues and the α-keto acid has long been suspected to be involved in this regulation [362,363]. A site-directed mutagenesis study led to the proposition of a model for the activation of Arabidopsis AOX by pyruvate [363]. In this model, the S-S involving Cys^78^ in both subunits would block the access of pyruvate to a reaction with this residue, whereas the reduction of the bond would allow the Cys^78^ thiol to interact with pyruvate. This is consistent with the increased AOX activity obtained after the substitution of Cys^78^ by Glu, which possesses a side chain able to mimic a thiohemiacetal [363,364]. This mutant is also insensitive to pyruvate [363]. However, the detection of a thiohemiacetal by mass spectrometry remains elusive in AOX and its crystal structure is not available [364]. Thus, although converging results suggest that AOX regulation by pyruvate occurs via the formation of a thiohemiacetal, the exact mechanism still remains to be elucidated.

## 14. Conclusions

The chemical properties of Cys residues make them reactive to a wide variety of PTMs. With this review, we wanted to cover the range of Cys PTMs present in plants. In some cases, for example, with the reversible formation of S-S, substantial research advances have been made over the years and it is now possible to provide an already broad overview of the involvement this process in plant biochemistry. For several other PTMs, such as persulfidation, *S*-cyanylation, *S*-glutathionylation, *S*-nitrosation, *S*-carbonylation, and CoAlation, we are still very much in the early steps in our exploration of their implication in the molecular physiology of plants. Future progress on these issues is undoubtedly going to benefit from current advances in specialized proteomic and chemistry techniques, the setting up of resources like specialized PTM databases, and the development of analytical and predictive bioinformatics tools. A point of particular interest is the fact that certain Cys residues can be modified by multiple PTMs under a variety of conditions (see, for example, the GAPDH catalytic Cys mentioned in this review). This exemplifies the sometimes complex interactions that can take place between different PTMs for a given Cys residue. We believe that we are currently only scratching the surface of this complexity.
